# AAV9 Gene Therapy for Ovarian Dysfunction and Reproductive Aging: Molecular Mechanisms, Therapeutic Targets, and Translational Perspectives

**DOI:** 10.3390/ijms27198516

**Published:** 2026-09-24

**Authors:** Safeer Ullah, Imran Ullah, Beshoy Sameh Fawzy Khalil, Zhaojun Geng, Jihyo Kim, Seung-Eun Lee, Xiaochun Wu, Kamran Saeed, Abdus Saboor Shah, Muhammad Idrees, Il-Keun Kong

**Affiliations:** 1 Division of Applied Life Science (BK21 Four), Gyeongsang National University, Jinju 52828, Gyeongnam Province, Republic of Korea; 2 Institute of Agriculture and Life Science, Gyeongsang National University, Jinju 52822, Gyeongnam Province, Republic of Korea; 3 Department of Biochemistry, Faculty of Biological Sciences, Quaid-i-Azam University, Islamabad 45320, Islamabad Capital Territory, Pakistan; 4 Biochemistry Research Laboratory, Department of Chemistry, GPGC Bannu, University of Science and Technology, Bannu 28100, Khyber-Pakhtunkhwa, Pakistan; 5 The King Kong Co., Ltd., Gyeongsang National University, Jinju 52828, Gyeongnam Province, Republic of Korea; 6 Division of Animal Science, Gyeongsang National University, Jinju 52828, Gyeongnam Province, Republic of Korea

**Keywords:** AAV9, ovarian dysfunction, female infertility, gene therapy, reproductive aging

## Abstract

Female infertility is a growing concern in developed countries as delayed childbearing and ovarian dysfunction become more common. Ovarian decline is characterized by follicular depletion, granulosa cell dysfunction, aneuploidy, spindle assembly defects, mitochondrial damage, DNA damage, telomere shortening, stromal remodeling, and cellular senescence. Current approaches (hormonal therapy, assisted reproductive technologies, platelet-rich plasma, stem cells, extracellular vesicles, and mitochondrial-targeted treatments) offer partial benefit but do not directly correct the molecular drivers of ovarian dysfunction. Gene therapy via viral vectors offers a more direct strategy. Adeno-associated virus serotype 9 (AAV9) is attractive for its broad tropism, long-term transgene expression, favorable safety profile, and evidence of ovarian somatic cell transduction, particularly in granulosa and interstitial cells. It remains experimental, however, and requires rigorous evaluation of biodistribution, immune response, off-target effects, germline safety, embryo development, offspring health, and tumorigenic risk. This review summarizes the mechanisms of ovarian dysfunction, current therapeutic limitations, AAV9’s advantages, delivery routes, candidate genes, and translational safety concerns.

## 1. Introduction

Ovarian dysfunction is a major cause of female infertility and is driven by follicular depletion, declining oocyte quality, granulosa cell dysfunction, mitochondrial damage, DNA damage, telomere attrition, inflammation, stromal remodeling, and cellular senescence [1,2]. At the cellular level, ovarian decline is multifactorial. Primordial follicles gradually decrease in number, reducing ovarian reserve and eventually causing reproductive senescence, but declining fertility is not due to follicle loss alone. Dysfunction also affects oocyte quality, granulosa cell function, mitochondrial activity, telomere maintenance, epigenetic stability, and the ovarian stromal microenvironment [1,2,3,4]. These defects contribute to poor fertilization, impaired embryo development, and aneuploidy. Genetic factors are also major contributors. According to the Human Infertility Disease Database (https://mdl.shsmu.edu.cn/IDDB/ (accessed on 3 August 2026)) and clinical studies, more than 300 genes are associated with human female fertility, of which about 136 are linked to the female infertility phenotype [5]. Current interventions, including assisted reproduction, hormonal support, antioxidants, mitochondrial supplements, platelet-rich plasma (PRP), stem cells, and extracellular vesicles, may improve selected outcomes but do not directly correct the molecular defects underlying ovarian decline.

Gene therapy is promising because it can deliver therapeutic genes to target cells and sustain modulation of decline-related pathways [6]. Adeno-associated virus (AAV) vectors have attracted attention for their favorable safety profile, low pathogenicity, ability to transduce dividing and non-dividing cells, and sustained transgene expression [7,8]. AAV vectors can penetrate the blood–follicle barrier and transduce ovarian granulosa cells, showing that the ovary is not inaccessible to viral gene delivery [7]. Functional studies show that viral gene delivery can rescue infertility caused by granulosa cell defects in mice, supporting the therapeutic targeting of ovarian somatic cells [7]. The viral capsid, delivery route, dose, species, age, and receptor environment of the target tissue primarily determine AAV tropism. Although AAV9 shows broad systemic biodistribution, recent capsid engineering studies demonstrate that AAV tropism can be redirected to specific organs, including the brain, heart, liver, and skeletal muscles [9,10,11,12]. This concept suggests that ovary-enriched capsids could be developed to improve delivery to reproductive tissues and specific ovarian compartments.

AAV9-mediated ovarian gene therapy also raises safety and translational concerns. Because AAV9 transduces multiple tissues, off-target expression must be carefully evaluated, especially with systemic delivery [8,13]. Successful translation will therefore require optimized delivery routes, promoter selection, controlled transgene expression, dose optimization, long-term safety analysis, fertility and offspring evaluation, and cancer risk assessment. This review aims to summarize the biological basis of ovarian dysfunction, evaluate current evidence for AAV9-mediated ovarian gene delivery, prioritize candidate genes, and discuss safety requirements for future translation.

## 2. Literature Search and Candidate Gene Selection Strategy

This study was conducted as a narrative review with a structured candidate gene selection strategy, rather than as a systematic review. Relevant genes and literature were identified using the Human Infertility Disease Database (IDDB), Online Mendelian Inheritance in Man (OMIM), ClinVar, GeneCards, PubMed, published diagnostic gene panels, and whole-exome sequencing studies. The literature and database searches were conducted from 2000 to 2026. Searches focused on female infertility, ovarian dysfunction, primary ovarian insufficiency, oocyte maturation defects, meiotic/DNA repair abnormalities, folliculogenesis, and reproductive endocrine disorders. Duplicate genes and genes with insufficient evidence for ovarian function or female infertility were excluded.

More than 300 infertility-associated genes were initially considered from available databases and published literature. Among these, 136 genes associated with female infertility in IDDB (https://mdl.shsmu.edu.cn/IDDB2020 (accessed on 3 August 2026)) were further evaluated. The exclusion criteria used to narrow it down from 300 to 136 were duplicates across databases and genes outside the scope of female infertility. Furthermore, genes with weak or indirect evidence, no clear ovarian relevance, and no function or clinical data were excluded, and approximately 63 genes with stronger evidence of ovarian dysfunction, oocyte defects, meiosis/DNA repair, endocrine dysfunction, or folliculogenesis were retained for further assessment. Finally, 11 genes were ultimately selected based on monogenic causality, patient recurrence, ovarian relevance, mechanistic clarity, non-lethal systemic phenotype, and animal model evidence. The complete gene categories and intermediate candidate lists are provided in Supplementary Data Tables S1–S7 and Supplementary Data Figure S1.

## 3. Genetic Causes of Ovarian Dysfunction

Female fertility depends on a large, coordinated genetic network spanning hypothalamic–pituitary endocrine signaling, gonadal development, germ cell specification, folliculogenesis, meiosis, oocyte maturation, fertilization, and early embryonic development [14]. With next-generation sequencing, curated resources and cohort studies have reported more than 300 genes associated with human infertility and reproductive function. In the human female infertility category, the Human Infertility Disease Database (IDDB) identified 136 genes, including 47 related to endocrine diseases, 60 to ovarian dysfunction, 10 to oocyte-factor infertility, and 19 to unspecified syndromes involving female infertility. From these 136 genes, we focused on the most biologically validated and clinically recurrent genes—those repeatedly reported in primary ovarian insufficiency, meiosis/DNA repair defects, oocyte maturation arrest, endocrine infertility, folliculogenesis defects, and diagnostic gene panels (Supplementary Data Table S1) [5]. This yielded approximately 63 major genes, from which we prioritized 11 for future AAV-based gene supplementation and feasibility analysis (Figure 1) based on monogenic causality, patient recurrence, ovarian relevance, mechanistic clarity, non-lethal systemic phenotype, and animal model evidence [5,15].

### 3.1. Primary Ovarian Insufficiency (POI)/Ovarian Failure Genes

POI and ovarian failure genes represent one of the most clinically important groups within the genetic causes of ovarian dysfunction [16,17]. These genes govern ovarian development, primordial follicle pool maintenance, oocyte survival, granulosa cell differentiation, oocyte–granulosa cell communication, and endocrine responsiveness (Supplementary Data Table S2). Pathogenic variants in this group are frequently reported in POI, diminished ovarian reserve, ovarian dysgenesis, and ovarian failure [16,17]. Many POI-related genes regulate the same processes that deteriorate during reproductive decline. For example, oocyte-derived factors such as *BMP15* and *GDF9* regulate granulosa cell proliferation and follicular growth, while transcription factors such as *NOBOX*, *FIGLA*, and *FOXL2* control oocyte identity, primordial follicle formation, and granulosa cell maintenance. Similarly, follicle-stimulating hormone receptor (*FSHR*) and *NR5A1* link genetic ovarian dysfunction to hormonal signaling and steroidogenesis, both of which are disrupted during reproductive decline [5,15,16,17].

### 3.2. Meiosis and DNA Repair Genes

Meiosis and DNA repair genes are essential for maintaining oocyte genomic integrity, homologous chromosome pairing, meiotic recombination, DNA double-strand break repair, chromosome synapsis, and sister chromatid cohesion [18]. Variants in this group are strongly associated with meiotic arrest, follicle loss, and POI (Supplementary Data Table S3) [15,17,19]. Genes such as *STAG3*, *REC8*, and *SMC1B* maintain meiotic cohesion, whereas *MCM8*, *MCM9*, *HFM1*, *MSH4*, and *MSH5* contribute to homologous recombination and DNA repair [5,15,17,19,20].

### 3.3. Oocyte Maturation and Fertilization Genes

Oocyte maturation and fertilization genes regulate the final steps of oocyte competence, including meiotic spindle assembly, cytoplasmic maturation, maternal mRNA regulation, polar body extrusion, fertilization, zygotic genome activation, and early embryonic development [21]. Defects commonly lead to oocyte maturation arrest, fertilization failure, or embryo arrest during assisted reproduction (Supplementary Data Table S4) [5,22]. Unlike POI genes, follicle numbers may be preserved; the major defect is the functional incompetence of oocytes. For example, *TUBB8* variants are strongly associated with abnormal meiotic spindle formation and oocyte maturation arrest, while *WEE2* is important for oocyte activation after fertilization. *PATL2* and *BTG4* are involved in maternal mRNA regulation and the oocyte-to-embryo transition, and *TLE6* is part of the subcortical maternal complex required for early embryonic development [5,15,23].

### 3.4. Hormonal Signaling and Endocrine Regulation Genes

Hormonal signaling and endocrine regulation genes control the hypothalamic–pituitary–gonadal axis, gonadotropin production, ovarian steroidogenesis, follicle recruitment, ovulation, luteinization, and feedback regulation [14,24]. Mutations in these genes can cause hypogonadotropic hypogonadism, anovulation, resistant ovarian syndrome, steroidogenic defects, abnormal puberty, primary amenorrhea, or endocrine-related infertility (Supplementary Data Table S5) [5,24]. Ovarian function depends on coordinated endocrine communication among the brain, pituitary, and ovary. *FSHR* and *LHCGR* regulate granulosa and theca cell responses to gonadotropins, while steroidogenic genes such as *STAR*, *CYP17A1*, *CYP19A1*, and *CYP21A2* are required for steroid hormone biosynthesis. Anti-Müllerian hormone (*AMH*) and *AMHR2* regulate follicle recruitment and ovarian reserve dynamics, whereas *KISS1/KISS1R*, *GNRH1/GNRHR*, and *TAC3/TACR3* participate in upstream reproductive axis activation and pulsatile gonadotropin control [5,15,24].

### 3.5. Folliculogenesis and Ovarian Development Genes

Folliculogenesis and ovarian development genes regulate germ cell specification, ovarian differentiation, primordial follicle formation, follicle activation, granulosa cell proliferation, ovulation, extracellular matrix remodeling, and zona pellucida formation [14]. Variants or dysregulation in this group may impair follicle establishment, follicle growth, ovulation, fertilization, or maintenance of the ovarian microenvironment (Supplementary Data Table S6) [14,17]. Ovarian development and folliculogenesis require interaction among germ cells, granulosa cells, stromal cells, endocrine signals, and extracellular matrix pathways. *BMPR2* participates in *BMP* signaling, which is important for follicle development and communication among ovarian cells. *WT1* and *PRDM1* contribute to gonadal and germ cell developmental programs, while *ZP3* is essential for zona pellucida structure and sperm–oocyte interaction. *HMMR* and *ALOX12* may influence granulosa cell proliferation, extracellular matrix dynamics, and ovulation-related signaling [5,15,17,25].

### 3.6. Regional Distribution and Occurrence of Human Female Infertility Genes

Regional analysis helps to prioritize genes for local diagnostic panels and select candidates for future gene supplementation studies. The selected regional pattern shows that East Asian studies repeatedly report genes related to POI, oocyte maturation, and DNA repair; Middle Eastern cohorts often highlight autosomal recessive meiotic and germ cell defects; European studies have historically emphasized POI and endocrine infertility; and United States cohorts reflect multi-ethnic gene panel and exome sequencing data (Figure 2 and Table 1). Across regions, three major biological pathways dominate the genetics of ovarian dysfunction: folliculogenesis and oocyte–granulosa signaling genes, such as *BMP15*, *GDF9*, *NOBOX*, and *FSHR*; meiosis and DNA repair genes, such as *STAG3*, *MCM8*, and *MCM9*; and oocyte maturation genes, such as *TUBB8* and *PATL2* (Figure 2 and Supplementary Data Table S7).

### 3.7. Biological Basis of Ovarian Dysfunction

Ovarian dysfunction disrupts multiple compartments of the oocyte, granulosa cells, follicular reserve, stromal microenvironment, vascular niche, and endocrine system (Figure 3). The ovarian reserve is established early in life and declines through follicular recruitment and atresia, while oocyte quality depends on mitochondrial function, meiotic spindle formation, chromosome segregation, DNA integrity, and cytoplasmic maturation [33,34]. Granulosa cells are also essential because they support follicle growth, steroidogenesis, metabolic exchange, and oocyte maturation by communicating closely with the oocyte [35,36]. Disruption of these processes can reduce ovarian reserve and cause impaired folliculogenesis, poor oocyte competence, abnormal steroidogenesis, ovulatory failure, embryo development defects, and infertility. Stromal fibrosis, vascular impairment, oxidative stress, inflammation, and endocrine decline further disturb the microenvironment and reduce reproductive potential.

## 4. Current Therapeutic Strategies for Ovarian Dysfunction

Current strategies mainly aim to preserve the remaining follicle pool, improve oocyte quality, reduce oxidative stress, support mitochondrial function, and enhance the ovarian microenvironment (Table 2). Lifestyle modification is among the simplest and safest approaches for slowing ovarian decline [37]. A healthy diet, regular physical activity, sleep regulation, weight control, and avoidance of smoking or environmental toxins may help reduce systemic inflammation and oxidative stress [2,37]. Lifestyle changes alone cannot restore lost follicles or reverse advanced decline; they are preventive or supportive rather than regenerative. Oxidative stress leads to granulosa cell apoptosis, poor oocyte maturation, meiotic spindle defects, telomere shortening, and reduced embryo developmental potential. Antioxidants have therefore been tested as therapies. Common candidates include melatonin, coenzyme Q10, *N*-acetylcysteine, resveratrol, vitamin C, vitamin E, alpha-lipoic acid, and polyphenols [38]. Antioxidant therapy is thus supportive, but insufficient to fully reverse ovarian decline. Many women with ovarian impairment or diminished reserve are managed with assisted reproductive technologies such as controlled ovarian stimulation, in vitro fertilization, and embryo or oocyte cryopreservation [39,40]. These do not reverse deterioration but may improve pregnancy chances by using the remaining follicle pool more efficiently.

Mitochondrial dysfunction is a central feature of ovarian dysfunction. Impaired oocytes show reduced ATP production, increased mitochondrial DNA damage, abnormal mitochondrial distribution, impaired calcium regulation, and higher oxidative stress [45]. Potential strategies include coenzyme Q10 supplementation, NAD^+^ precursor therapy, mitochondrial antioxidants, mitophagy regulation, and mitochondrial transfer approaches [3,46,47]. However, these techniques raise technical, ethical, safety, and regulatory concerns and primarily target oocyte quality rather than the broader ovarian environment—including stromal fibrosis, inflammation, follicle depletion, and endocrine decline. Platelet-rich plasma (PRP) has recently gained attention as a possible ovarian rejuvenation strategy. Stem-cell-based therapy is another promising regenerative approach for ovarian deterioration and premature ovarian insufficiency [49]. Stem cell therapy still faces major challenges: cell source variability, dose and delivery optimization, long-term safety, possible tumor risk, immune response, and a lack of standardized protocols.

## 5. Rationale for Using AAV9 in Ovarian Gene Therapy

Adeno-associated virus serotype 9 (AAV9) is among the most attractive vectors for in vivo gene therapy, combining broad tropism, low immunogenicity, long-term transgene expression, and transduction of both dividing and non-dividing cells (Table 3) [52]. These traits suit ovarian gene therapy, in which the target organ contains multiple cell types, including oocytes, granulosa cells, theca cells, stromal cells, endothelial cells, immune cells, and surface epithelial cells.

### 5.1. Basic Features of AAV Vectors

Adeno-associated viruses are small, non-enveloped, single-stranded DNA viruses belonging to the *Parvoviridae* family [52]. AAV vectors are widely used in gene therapy because they are generally considered non-pathogenic, replication defective, and capable of supporting stable transgene expression in many tissues [8,52]. Most viral coding sequences are removed and replaced with a therapeutic cassette, while the inverted terminal repeats are retained for genome packaging and vector production. AAV vectors are noted for their low pathogenicity, low immunogenicity, broad tropism, and sustained expression, explaining their prominence in gene therapy research [8,52].

A key advantage of AAV vectors is the transduction of both dividing and non-dividing cells. AAV vectors persist mainly as episomal DNA rather than integrating, reducing the risk of insertional mutagenesis relative to integrating vectors, though episomal genomes may be diluted in rapidly dividing cells. A key limitation is restricted packaging capacity. The effective AAV genome capacity is generally around 4.7 kb, although some studies discuss a range of approximately 4.7–5.0 kb [8,52]. This is a major design consideration because the cassette must fit the promoter, transgene, regulatory elements, and polyadenylation signal within a limited space. For large genes, optimization is essential: compact promoters, removal of non-essential regulatory sequences, shorter polyA signals, or minimized transgene designs.

### 5.2. Biological Advantages of AAV9

AAV9 has several features useful for ovarian and reproductive research. First, it has broad in vivo tropism and is widely used for systemic delivery; after systemic administration, it transduces the liver, heart, skeletal muscle, central nervous system, and other tissues, depending on dose, species, age, route, and promoter [8,53]. Second, AAV9 supports sustained transgene expression. Ovarian decline is not acute but progressive, with long-term loss of follicle quality, mitochondrial activity, genome stability, endocrine function, and microenvironment. A vector that maintains expression over weeks to months is therefore better suited than a short-acting drug. Third, AAV9 has relatively low pathogenicity and immunogenicity compared with other viral vectors [52]. This matters because reproductive tissues require careful safety evaluation: ovarian gene therapy must consider inflammation, off-target transduction, germline exposure, reproductive outcomes, and effects on offspring. AAV9 does not eliminate these risks but, given its established use, provides a strong basis for the rational development of ovarian vectors.

### 5.3. Evidence for Ovarian Transduction

Direct experimental evidence provides a serotype-specific rationale for considering AAV9 in ovarian gene delivery. Kanatsu-Shinohara et al. compared multiple AAV serotypes, including AAV1, AAV6, AAV6.2, AAV7M8, AAV9, AAV-DJ, and AAV-DJ8, following ovarian administration. Among the tested vectors, AAV9 produced the strongest transduction signal in granulosa cells and was therefore selected for subsequent experiments. AAV9 was able to penetrate the blood–follicle barrier and transduce ovarian somatic cells without detectable reporter expression in oocytes [7]. AAV9 has been highlighted as one of the strongest serotypes for granulosa cell transduction. More recent work comparing AAV8 and AAV9 further showed that both vectors can deliver transgenes predominantly to granulosa and interstitial cells [56]. Thus, current evidence does not establish AAV9 as uniquely superior to all AAV serotypes; rather, it supports AAV9 as one of the experimentally validated candidate platforms for ovarian somatic cell targeting. Further comparative studies and capsid optimization will be required to establish an ovary-enriched vector.

### 5.4. Possible Delivery Routes for Ovarian Targeting

Several delivery routes may be considered for AAV9-mediated ovarian gene therapy, each with specific advantages and limitations.

#### 5.4.1. Direct Intraovarian Injection

Direct ovarian injection is among the most targeted approaches, placing a vector into or near ovarian tissue to increase local transduction and reduce systemic exposure [7]. It is especially useful for proof-of-concept studies testing whether a therapeutic gene improves ovarian decline markers, follicle reserve, or reproductive outcomes.

#### 5.4.2. Intravenous/Systemic Delivery

Intravenous (IV) delivery enables broad tissue distribution and facilitates the study of ovarian decline in systemic dysfunction, given AAV9’s systemic reach and broad tropism [57]. It may target the ovary, as well as the liver, heart, brain, kidney, muscle, and immune tissues. However, because the ovary is small and AAV9 accumulates in highly perfused tissues, few vectors may reach it after IV delivery. IV delivery thus suits systemic studies, whereas direct ovarian or periovarian delivery is preferable for ovary-focused therapy.

#### 5.4.3. Intraperitoneal Delivery

Intraperitoneal (IP) injection is well suited to small-animal studies, as it is technically easier than direct ovarian or tail vein injections. Because the ovaries lie in the peritoneal cavity, IP delivery may indirectly expose them to the vector; it is useful for pilot studies but less precise than direct ovarian delivery.

#### 5.4.4. AAV9 Tropism Engineering

Tropism is determined mainly by the AAV capsid, delivery route, dose, species, age, and target-cell receptor environment. Importantly, AAV tropism can be redirected. For example, brain-directed AAV9 variants have been engineered to improve blood–brain barrier penetration and central nervous system transduction after systemic administration [9,61]. Similarly, muscle-directed AAV variants have been designed or selected to enhance skeletal and cardiac muscle transduction while reducing liver accumulation [12,62]. Cardiac-directed capsid engineering has also generated myocardium-tropic AAV variants with improved cardiac infectivity and reduced liver transduction, supporting the feasibility of redirecting AAV vectors toward the heart [10]. In parallel, liver-directed AAV engineering has produced AAV3B- and AAV7/AAV8-derived variants with enhanced human hepatocyte tropism, demonstrating that capsid modification can also enhance organ-selective delivery to hepatocytes [11]. These examples provide a framework for ovarian AAV engineering: if capsid–receptor interactions can be tuned for brain or muscle tropism, a similar strategy could enrich delivery to ovarian cells (Figure 4).

### 5.5. Vector Design Considerations for Ovarian Gene Therapy

Successful AAV9 ovarian gene therapy depends on cassette design. Because packaging capacity is limited, every element must be chosen carefully. The promoter must drive therapeutic expression without excessive off-target activity: ubiquitous promoters (CMV and CAG) give strong expression, while ovary- or cell-type-specific promoters improve targeting and safety [7,8]. The promoter choice should match the biological goal: a strong general promoter for broad ovarian support in preclinical studies or a targeted promoter for granulosa-cell-specific modulation [7,67]. For the 11 prioritized genes, promoter choice should match the target cell type and gene function (Figure 5). Oocyte-centered genes (*BMP15*, *GDF9*, *NOBOX*, *TUBB8*, *PATL2*, *FIGLA*, *SOHLH1*) pair best with oocyte or germ cell promoters such as *GDF9*, *ZP3*, *FIGLA*, *OOG1*, *SOHLH1*, or *DDX4/VASA* [68,69,70,71]. Among these, *GDF9* and *ZP3* promoters suit growing oocyte expression; *FIGLA* suits early oocyte/primordial follicle genes; and *SOHLH1* is especially relevant to germ cell and early oocyte differentiation [69,71,72]. For granulosa or somatic cell genes (*FSHR*, *FOXL2*, *NR5A1*), granulosa/steroidogenic promoters such as *FSHR*, *AMH/AMHR2*, *FOXL2*, or *NR5A1/SF-1* may give better cell-type restrictions [67,73]. For meiotic and DNA repair genes such as *MCM8*, germ cell or oocyte-stage-specific promoters (*DDX4/VASA*, *GDF9*, *ZP3*, *OOG1*, and *SOHLH1*) are preferable, depending on whether expression is needed in early germ cells, growing oocytes, or later oocytes [68,72,74,75]. Promoter choice should thus vary by gene class: oocyte-directed promoters for secreted oocyte–granulosa signaling genes, granulosa or steroidogenic promoters for granulosa/endocrine response genes, and germ cell or oocyte-stage-specific promoters for meiotic/DNA repair genes. This improves specificity, reduces off-target expression, and increases safety. Regulatory elements such as the Woodchuck Hepatitis Virus Posttranscriptional Regulatory Element (WPRE) enhance expression but consume cassette space, so for large genes they may need shortening or removal to stay within the packaging limit.

## 6. Candidate Genes for AAV9-Mediated Ovarian Rejuvenation

*BMP15* is among the strongest ovary-centered candidates: produced mainly by the oocyte, it acts on granulosa cells to coordinate follicle growth, cumulus expansion, steroidogenic balance, and oocyte competence [76,77]. *GDF9*, an essential oocyte-secreted member of the TGF-β superfamily, is a central regulator of early folliculogenesis. BMP15 forms the core oocyte-to-granulosa signaling axis that governs granulosa cell proliferation, cumulus function, follicle survival, and oocyte competence [77]. *NOBOX* is an oocyte-specific homeobox transcription factor required for early follicle formation, oocyte gene programming, and maintenance of the primordial follicle pool, regulating downstream genes for zona pellucida formation, oocyte growth, and follicular survival [15,17]. *TUBB8*, an oocyte-enriched beta-tubulin gene, is critical for meiotic spindle assembly, chromosome alignment, polar body extrusion, and oocyte maturation. Pathogenic variants in *TUBB8* are a leading cause of oocyte maturation arrest and IVF failure, particularly in East Asian studies [78]. *FSHR* encodes the receptor through which follicle-stimulating hormone acts on granulosa cells to regulate follicle growth, aromatase expression, estradiol production, and antral follicle development. Loss- or altered-function *FSHR* variants are associated with ovarian dysgenesis, resistant ovary syndrome, primary amenorrhea, poor ovarian response, and altered ovarian reserve [79]. *MCM8*, a DNA repair and homologous recombination factor, is important for meiotic genome stability and for maintaining ovarian reserve. Pathogenic *MCM8* variants are associated with POI, gonadal dysgenesis, chromosomal instability, and infertility [20]. *PATL2*, an oocyte-expressed RNA-binding and translational regulation gene, is required for oocyte maturation and early embryonic competence [80]. *FIGLA* is an oocyte-specific basic helix-loop-helix transcription factor required for primordial follicle formation and regulation of oocyte genes, including zona pellucida genes. It acts at a key checkpoint—the transition from germ cell cysts to primordial follicles and establishment of the early follicle pool [81]. *FOXL2* is a forkhead transcription factor required for ovarian differentiation, granulosa cell identity, follicle maintenance, and prevention of testis-like differentiation in the ovary [82]. *NR5A1* (steroidogenic factor 1) regulates adrenal and gonadal development, steroidogenesis, reproductive endocrine function, and the expression of genes required for ovarian hormonal activity. Human *NR5A1* variants are reported in disorders of sex development, ovarian insufficiency, poor ovarian response, and endocrine-related reproductive dysfunction [83]. *SOHLH1* is a germ cell transcription factor involved in early oocyte differentiation, primordial follicle activation, and the network that prepares oocytes for follicular development. It interacts with other early germ cell regulators and helps drive the transition from primordial germ cell/oocyte programs toward follicle-stage competence [72]. AAV packaging feasibility depends on the total expression cassette size, including the CDS, promoter, polyadenylation signal, and optional regulatory elements. For relatively larger genes such as FSHR and MCM8, compact promoters and polyadenylation signals, and the omission of non-essential elements such as WPRE can be used to maintain compatibility with AAV packaging capacity (Table 4).

## 7. Mechanisms by Which AAV9 Gene Therapy May Improve Ovarian Impairment

### 7.1. Restoration of Telomere Maintenance and Genomic Stability

One major mechanism of ovarian aging is telomere shortening and accumulation of DNA damage [58]. With age, ovarian somatic cells, including granulosa and stromal cells, lose proliferative and repair capacity. Short telomeres activate DNA damage responses, drive p53/p21- and p16-related senescence, and reduce granulosa cell support of the oocyte [33,84]. AAV9 delivery of genes such as *TERT* may restore telomere maintenance and reduce age-associated DNA damage. In aged mice, AAV9 telomerase therapy delayed aging and improved healthspan without increasing cancer incidence, with benefits linked not only to telomere biology but also to improved metabolic and mitochondrial fitness [57]. In the ovary, improved genomic stability in granulosa, stromal, and possibly surface epithelial cells could create a healthier microenvironment for follicle development; by reducing DNA damage signaling and senescence, *AAV9-TERT* or similar therapy may support follicular survival and oocyte competence.

### 7.2. Reduction of Oxidative Stress

Oxidative stress is a central driver of ovarian deterioration. Aged ovaries show increased reactive oxygen species, reduced antioxidant defense, mitochondrial damage, lipid peroxidation, and impaired oocyte–granulosa communication [84]. AAV9 gene therapy may help by delivering genes that enhance antioxidant defense or reduce ROS, including *TERT*, *SIRT* family genes, *FOXO* family genes, antioxidant enzymes, mitophagy regulators, and other mitochondrial protective factors. Reducing mitochondrial dysfunction would lower ROS, protecting oocytes and granulosa cells from oxidative injury [85]. This matters because follicles are highly sensitive to oxidative stress: even moderate damage can disturb folliculogenesis, steroidogenesis, meiotic spindle organization, and mitochondrial distribution.

### 7.3. Improvement of Mitochondrial Function and Energy Metabolism

Mitochondrial dysfunction is another key feature. Oocytes require abundant ATP for spindle formation, chromosome segregation, cytoplasmic maturation, fertilization, and early embryonic development. With age, mitochondrial DNA damage, reduced ATP production, abnormal mitochondrial distribution, and increased ROS production contribute to poor oocyte quality [86]. Recent reviews describe mitochondrial dysfunction as a core mechanism linking oxidative stress, metabolic reprogramming, and reproductive decline during ovarian decline [87]. AAV9 may improve mitochondrial function by delivering genes that enhance biogenesis, mitophagy, antioxidant capacity, or metabolic homeostasis—for example, *PGC-1α* signaling, *SIRT* pathways, mitophagy regulators, or telomerase-associated mitochondrial protection [87].

### 7.4. Suppression of Cellular Senescence and SASP

Cellular senescence is a major contributor. Aged granulosa and stromal cells become senescent through telomere shortening, DNA damage, oxidative stress, mitochondrial dysfunction, and inflammatory signaling [4]. SASP factors disrupt the microenvironment by increasing inflammation, matrix remodeling, and fibrosis and by inflicting paracrine damage on nearby follicles [4]. AAV9 may reduce senescence by delivering genes that improve DNA repair, telomere stability, mitochondrial quality, or anti-inflammatory signaling; *AAV9-TERT*, for instance, may act through improved telomere maintenance and reduced DNA damage signaling, while other genes may lower *p16*, *p21*, *NF-κB* activation, or SASP cytokines.

## 8. Preclinical Evidence Supporting AAV9 Ovarian Gene Therapy

### 8.1. Evidence That AAV Vectors Can Transduce Ovarian Tissue

Preclinical studies show AAV vectors can deliver genes into ovarian tissue: one demonstrated that AAVs penetrate the blood–follicle barrier and infect granulosa cells, which regulate follicle growth, steroidogenesis, oocyte maturation, and follicular survival [7]. A strong example comes from a congenital infertility mouse model: Kanatsu-Shinohara and colleagues showed that AAV-mediated delivery of the *Kitl* gene restored oogenesis in *Kitl*-mutant infertile mice. This demonstrates a clear principle: an ovarian genetic or molecular defect can be corrected by viral gene delivery, improving reproduction. Although the model is congenital infertility rather than natural decline, it shows ovarian function can be therapeutically modified by AAV gene supplementation (Table 5) [7].

### 8.2. Evidence for Granulosa Cell Targeting

The preclinical AAV studies in the ovary showing granulosa cell infection are therefore directly relevant to ovarian dysfunction [7]. If AAV vectors reach granulosa cells, genes such as *BMP15*, *FSHR*, *SIRT* genes, antioxidant genes, or mitochondrial regulators could be delivered to improve granulosa cell survival and function. For defects primarily involving granulosa cells or the follicular microenvironment, direct oocyte transduction may be unnecessary, as oocyte competence strongly depends on granulosa cell support. However, granulosa cell targeting cannot directly correct oocyte-intrinsic genetic defects affecting meiosis, spindle assembly, or maternal effect functions.

### 8.3. Evidence from AAV9-Mediated Anti-Aging Gene Therapy

AAV9 has been used successfully in aging studies. Systemic AAV9 delivery of mouse *TERT* improved several aging outcomes in adult and old mice—insulin sensitivity, osteoporosis, neuromuscular coordination, and molecular aging markers—with increased longevity and no increased cancer incidence under the conditions tested [57]. For ovarian aging, *AAV9-TERT* could reduce telomere-associated DNA damage, senescence, mitochondrial dysfunction, and tissue degeneration. Because ovarian and systemic decline share oxidative stress, inflammation, mitochondrial decline, and senescence, AAV9 anti-aging therapy offers a logical foundation for ovarian rejuvenation studies.

### 8.4. Evidence from AAV9 in Reproductive and Ovarian Disease Models

AAV9 has also been used in reproductive disease models: AAV9 delivery of modified Müllerian inhibiting substance was therapeutic in mouse ovarian cancer, showing AAV9 can produce meaningful effects in ovarian disease models [59]. Although ovarian cancer and dysfunction differ, this shows AAV9 can deliver therapeutic proteins with measurable in vivo effects in female reproductive models. In ovarian deterioration, the goal would shift from suppressing tumor growth to improving follicle survival, reducing senescence, restoring homeostasis, and enhancing reproduction.

### 8.5. Biological Limitations and Critical Feasibility Considerations

Ovarian dysfunction and reproductive aging are multifactorial processes; therefore, a single-gene supplementation strategy is unlikely to reverse all age-associated defects [1]. Gene therapy may be most effective when a dominant molecular defect or well-defined monogenic cause is present [15]. For complex ovarian aging, therapeutic benefit may require targeting shared upstream pathways, employing combination strategies, or targeting genes that improve multiple cellular processes [1].

The optimal target cell also depends on the underlying defect. Granulosa cell transduction may improve follicular signaling, steroidogenesis, metabolism, and oocyte support [35], but it cannot directly correct oocyte-intrinsic mutations affecting meiosis, spindle assembly, or maternal effect genes. Conversely, direct oocyte transduction raises substantially greater germline safety concerns [93]. In addition, incomplete transduction, heterogeneous ovarian cell populations, follicular turnover, and loss of vector genomes in dividing cells may restrict the magnitude and duration of therapeutic benefit [94]. These limitations should be considered when selecting candidate genes, target cells, and delivery strategies.

## 9. Safety Concerns and Translational Challenges

### 9.1. Immune Responses Against AAV9 Vectors

One of the major limitations of AAV-based gene therapy is the host immune response against the viral capsid or the therapeutic transgene [54,95]. AAV vectors are less immunogenic than adenoviral or lentiviral vectors, but not non-immunogenic. Many people have been exposed to wild-type AAVs and carry pre-existing neutralizing antibodies, which can reduce transduction, alter biodistribution, and sometimes increase inflammatory responses after delivery [54,95]. This is especially relevant for systemic AAV9, since circulating antibodies may neutralize the vector before it reaches the target. Beyond humoral immunity, cellular responses can eliminate transduced cells expressing capsid peptides or a foreign transgene [54,95]. This may reduce durability and, if clearance occurs in the ovary, harm ovarian cells, since inflammation can affect follicular viability, stromal function, vascular integrity, and endocrine secretion.

### 9.2. Dose-Related Systemic Toxicity

AAV safety depends on dose, route, tropism, and patient health. High-dose systemic therapy has been linked to hepatotoxicity, complement activation, thrombocytopenia, thrombotic microangiopathy, and neurotoxicity. Despite several approved rAAV products, high-dose systemic administration is still associated with immune toxicity, liver damage, genotoxicity, thrombotic microangiopathy, and neurological adverse effects [8,95]. Dose optimization and route selection will therefore be pivotal to safe development.

### 9.3. Off-Target Biodistribution and Non-Ovarian Transduction

Because of its broad tropism, AAV9 transduces liver, heart, skeletal muscle, nervous tissue, and reproductive organs [8,53]. This is useful for systemic studies but poses an off-target risk: unintended activation of rejuvenation genes in non-target organs may have unforeseen consequences, especially for genes governing cell survival, proliferation, extracellular matrix remodeling, telomere maintenance, metabolism, or inflammation. This is significant for candidate genes, which may benefit senescent ovarian tissue but disrupt homeostasis if expressed unregulated elsewhere. Ovarian gene therapy should therefore use tissue-selective promoters, local delivery, dose restriction, or regulated expression to limit off-target activity.

### 9.4. Germline and Reproductive Safety

Germline safety is a major concern for ovarian AAV gene therapy because the ovary contains both somatic support cells and germ cells. Available AAV studies in ovarian tissue indicate that somatic compartments, particularly granulosa cells, can be efficiently transduced [7]. In the study by Kanatsu-Shinohara et al., AAV-mediated ovarian delivery restored fertility in infertile mice, and reporter expression was primarily observed in granulosa cells rather than oocytes, supporting the feasibility of targeting somatic ovarian cells while limiting direct germ cell transduction under these experimental conditions [7]. However, this finding should not be interpreted as proof that germline exposure can be excluded in all settings, because vector distribution depends on capsid, dose, route, species, age, and tissue environment [7].

Recent evidence further emphasizes this concern. Assaf and DeKeyser detected AAV vector genome and transgene mRNA in oocytes of a female cynomolgus macaque 4 weeks after systemic intravenous administration of an engineered rAAV vector, demonstrating that oocyte exposure can occur under certain conditions [93]. Regulatory and preclinical literature therefore recommends careful evaluation of gonadal biodistribution, germ cell transduction, reproductive toxicity, and the possibility of vertical transmission [60]. Accordingly, future ovarian AAV studies should directly assess vector genomes and transgene expression in isolated oocytes, embryos, and offspring and should evaluate fertility, pregnancy outcomes, embryonic development, offspring health, and potential germline transmission before clinical translation is considered.

### 9.5. Genotoxicity and Insertional Mutagenesis

AAV vectors persist mainly as episomal DNA, a reason they are safer than integrating vectors; still, rare integration is possible and should be evaluated in long-term studies [55]. Although episomal persistence dominates, both wild-type and recombinant AAV can integrate into host DNA, and the long-term consequences require careful evaluation [8,55]. This matters for long-lived tissues and treatments seeking sustained expression. Ovarian therapy may require prolonged exposure to maintain follicular, stromal, vascular, or endocrine benefit, necessitating long-term monitoring for rare genotoxic events.

### 9.6. Route of Delivery and Clinical Feasibility

The ideal delivery route is yet to be determined. Intravenous injection is clinically appealing because of its minimally invasive nature and suitability for systemic reproductive decline; yet, extensive AAV9 biodistribution heightens off-target exposure. Consequently, intravenous delivery must be integrated with ovary-targeted capsid tropism, cell-specific promoters, and dosage modification to enhance ovarian selectivity and clinical viability.

### 9.7. Liver Toxicity and Systemic Safety

The liver is heavily exposed to AAV after systemic administration; enzyme elevation, immune-mediated injury, and severe hepatotoxicity have been reported [8,95]. Liver safety should be monitored with alanine aminotransferase (ALT), aspartate aminotransferase (AST), bilirubin, histology, inflammatory markers, and vector biodistribution. Direct ovarian delivery, by reducing the total dose, may lower systemic exposure.

### 9.8. Regulatory and Regional Translational Considerations

Beyond vector-specific safety, the clinical translation of AAV-mediated ovarian gene therapy will depend on regional regulatory requirements for gene therapy products. Major regulatory authorities, including the U.S. Food and Drug Administration (FDA), European Medicines Agency (EMA), Medicines and Healthcare products Regulatory Agency (MHRA), Pharmaceuticals and Medical Devices Agency (PMDA), Ministry of Food and Drug Safety (MFDS), National Medical Products Administration (NMPA), and Therapeutic Goods Administration (TGA), generally require rigorous assessment of vector quality, non-clinical pharmacology and toxicology, biodistribution, dose justification, immunogenicity, and long-term safety [96,97,98,99,100,101]. For ovarian-directed AAV therapy, these requirements are particularly important because intended transduction of ovarian somatic cells must be distinguished from unintended exposure of oocytes or other germ cell populations. Consequently, reproductive toxicity, vector persistence, fertility and pregnancy outcomes, offspring health, and potential germline transmission should be incorporated into the preclinical development strategy. Regional regulatory considerations relevant to ovarian-directed AAV therapy are summarized in Table 6.

## 10. Future Perspectives

Future AAV9-mediated ovarian gene therapy should prioritize enhancing ovarian selectivity and minimizing off-target transduction. AAV9 exhibits extensive tropism and can transduce ovarian somatic cells; nevertheless, its dissemination to the liver, heart, muscle, and other organs raises safety concerns. Consequently, subsequent research should focus on ovary-enriched capsid engineering, optimal delivery mechanisms, cell-specific promoters, and regulated transgene expression. Engineering ovarian tropism is a primary objective. Modification of the capsid, peptide insertion, directed evolution, or receptor-mediated screening may facilitate the development of AAV variants with enhanced ovarian uptake and reduced hepatic accumulation. Ovary-targeted vectors would be particularly advantageous for systemic delivery, as the existing AAV9 biodistribution restricts ovarian selectivity. The selection of promoters is equally significant. Robust ubiquitous promoters such as CMV or CAG are advantageous for initial proof-of-concept investigations but may increase off-target expression. Subsequent research should employ ovary- or cell-specific promoters aligned with gene function. Genes associated with oocytes, including *BMP15*, *GDF9*, *NOBOX*, *TUBB8*, *PATL2*, *FIGLA*, and *SOHLH1*, may be coupled with the promoters of *GDF9*, *ZP3*, *FIGLA*, *OOG1*, *SOHLH1*, or *DDX4/VASA* [68,70,71]. Granulosa or steroidogenic genes, including *FSHR*, *FOXL2*, and *NR5A1*, may be more effectively regulated by the promoters of *FSHR*, *AMH/AMHR2*, *FOXL2*, or *NR5A1/SF-1* [67,73]. Targeting granulosa cells is particularly advantageous, as they facilitate follicle development, steroidogenesis, oocyte maturation, and maintenance of the ovarian microenvironment. Direct intraovarian delivery may be beneficial for preclinical validation; however, clinical application may necessitate less invasive, image-guided, intra-bursal, catheter-based, or ovary-targeted systemic methods. Subsequent research must functionally validate candidate genes and evaluate biodistribution, germline safety, embryonic development, progeny health, immunological responses, off-target effects, and tumorigenic risk.

Translational development of ovarian-directed AAV gene therapy will also require sustained financial support and multidisciplinary collaboration. Although few funding programs are specifically dedicated to ovarian AAV gene therapy, several governmental and philanthropic organizations support related areas, including reproductive biology, ovarian aging, women’s health, gene therapy, regenerative medicine, and advanced therapeutic development. Representative funding agencies and programs across major geographic regions are summarized in Supplementary Data Table S8. These funding mechanisms may support different stages of development, ranging from target validation and vector engineering to preclinical biodistribution, safety assessment, and translational studies.

## 11. Conclusions and Limitations

AAV9-mediated ovarian gene therapy remains an experimental approach rather than an established treatment for ovarian dysfunction. Current proof-of-concept evidence demonstrates that AAV vectors can transduce ovarian somatic cells and that gene supplementation can restore ovarian function in selected genetic models. Comparative screening has also identified AAV9 as a candidate serotype for granulosa cell transduction. However, these findings are limited to specific preclinical models and do not establish AAV9 as uniquely superior, ovary-specific, or effective against multifactorial ovarian aging. Most candidate genes discussed in this review have not yet been functionally validated using ovarian AAV9 delivery. Future studies should directly compare AAV serotypes, validate individual gene targets and target cell populations, optimize capsids, promoters, delivery routes, and doses, and determine the durability of therapeutic effects. Rigorous assessment of biodistribution, immune responses, off-target effects, tumorigenic risk, oocyte exposure, germline transmission, embryo development, and offspring health will also be essential. Therefore, AAV9 should currently be regarded as an experimental candidate platform for ovarian somatic-cell gene delivery that requires substantial validation before clinical translation.

## Figures and Tables

**Figure 1:**
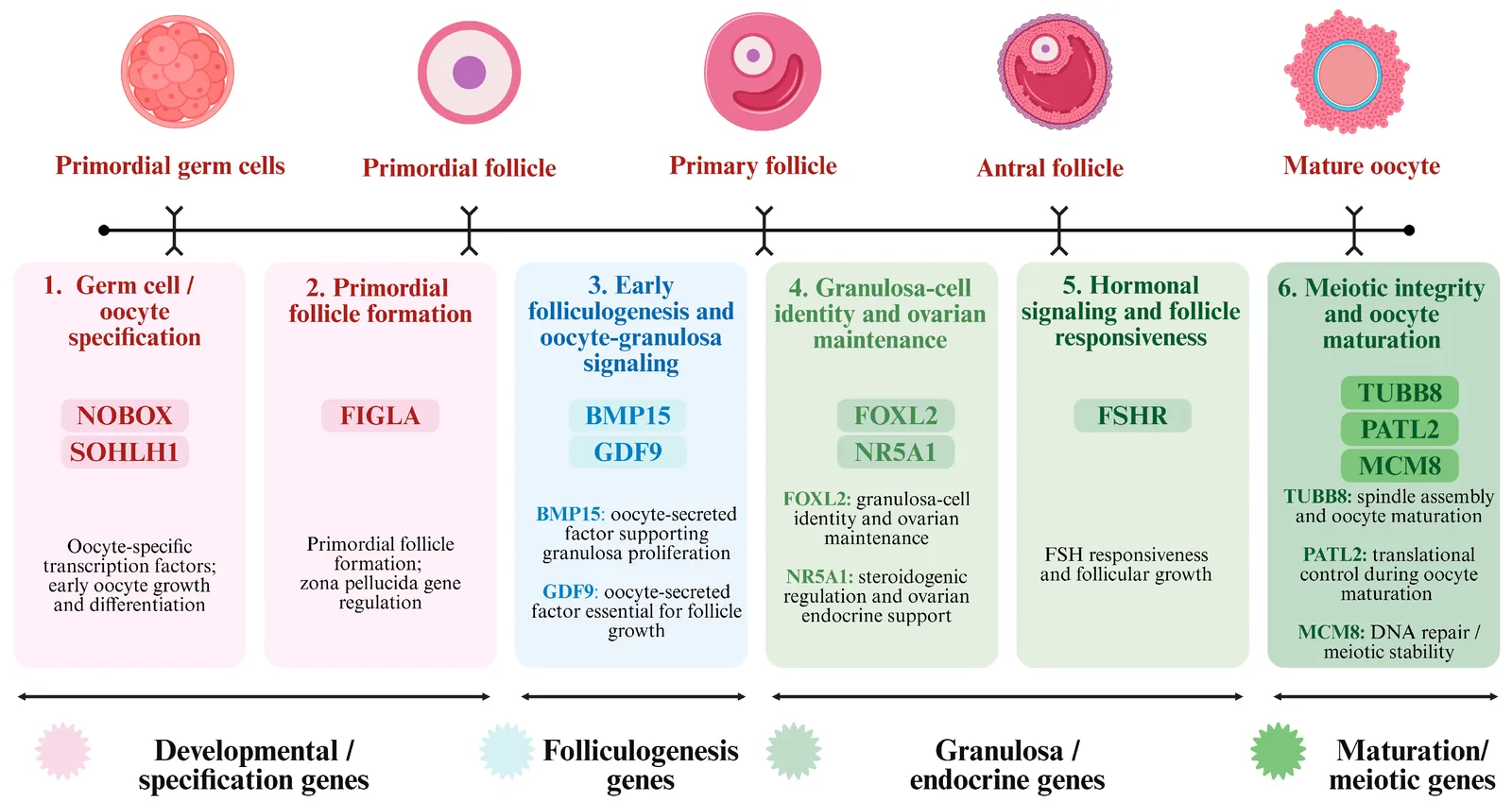
Functional roles of prioritized ovarian genes. Major biological functions of selected candidate genes in ovarian dysfunction: follicle formation, oocyte–granulosa communication, granulosa cell identity, endocrine regulation, meiosis, DNA repair, spindle formation, oocyte maturation, and fertility maintenance. Created with BioRender.com.

**Figure 2:**
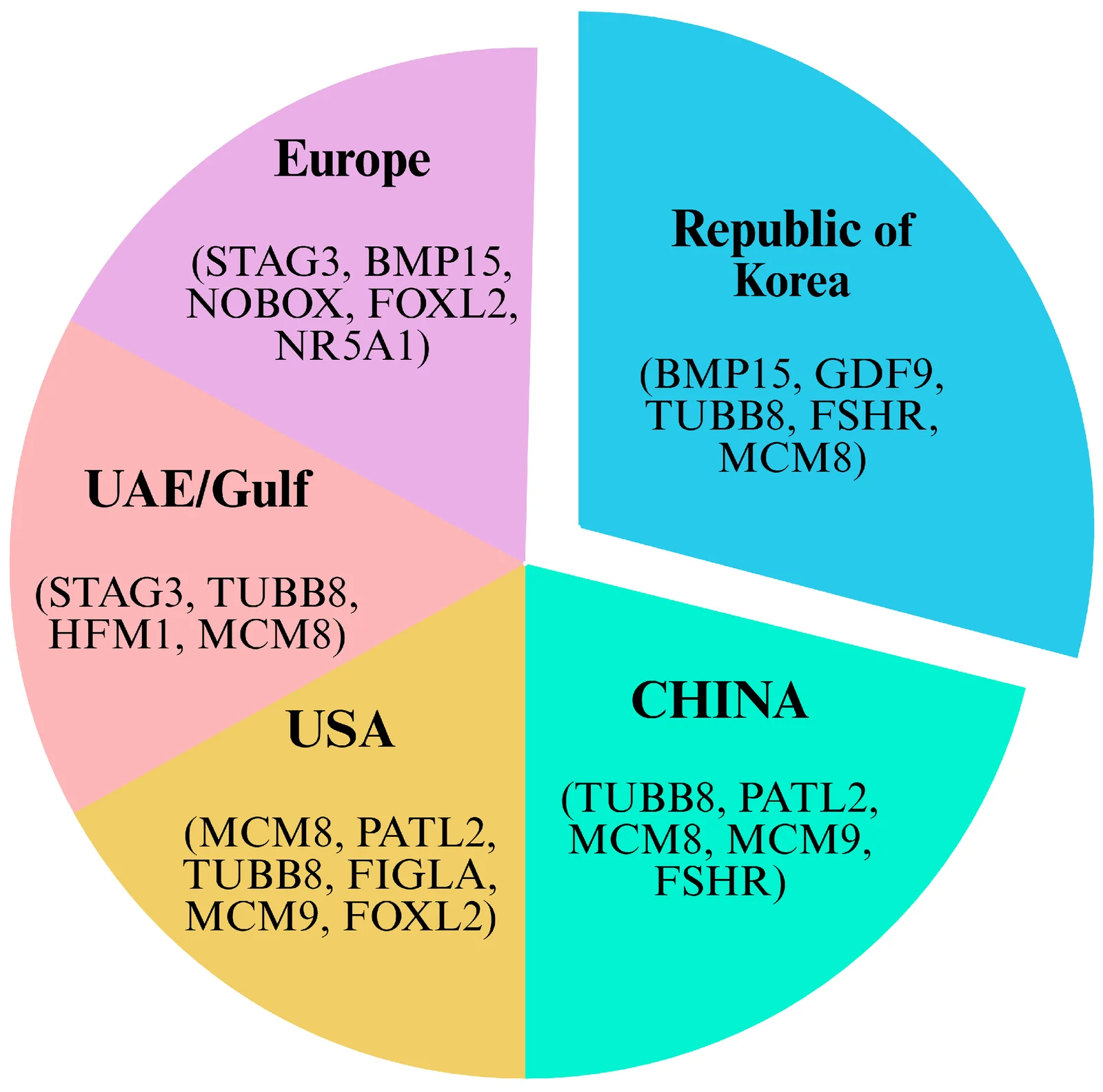
Region-specific prioritization of candidate ovarian genes. The figure summarizes selected candidate genes associated with ovarian dysfunction across different regions, including the Republic of Korea, China, the USA, the UAE/Gulf, and Europe. Created with BioRender.com.

**Figure 3:**
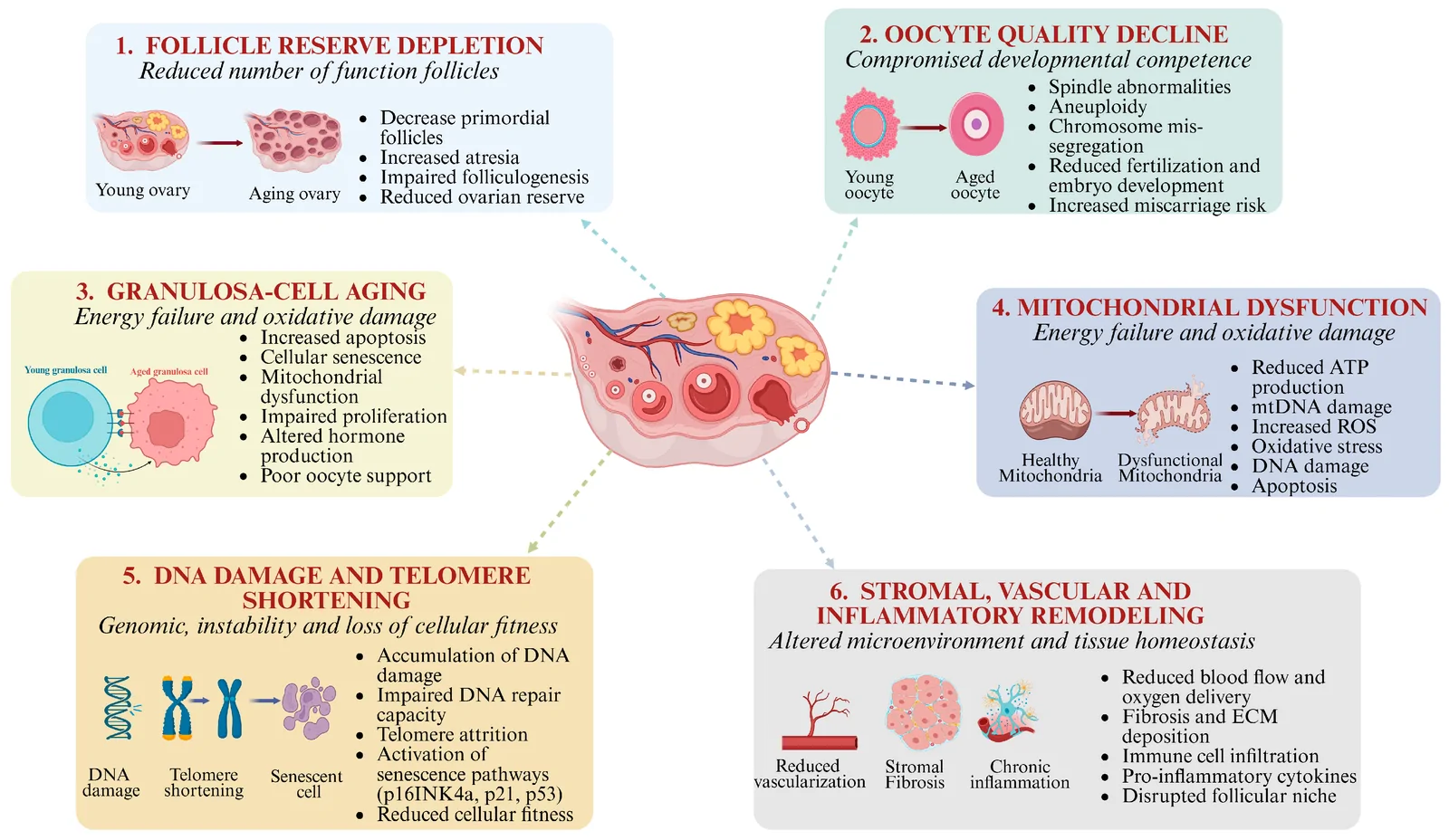
Major cellular and molecular mechanisms of ovarian dysfunction. Key drivers of ovarian decline include depletion of the follicle reserve, declining oocyte quality, granulosa cell deterioration, mitochondrial dysfunction, oxidative stress, DNA damage, telomere shortening, stromal remodeling, vascular decline, inflammation, and senescence. Together, these processes impair ovarian function and fertility. Created with BioRender.com. The colored boxes distinguish the six major mechanism categories, while dashed arrows connect the central ovary to each associated mechanism

**Figure 4:**
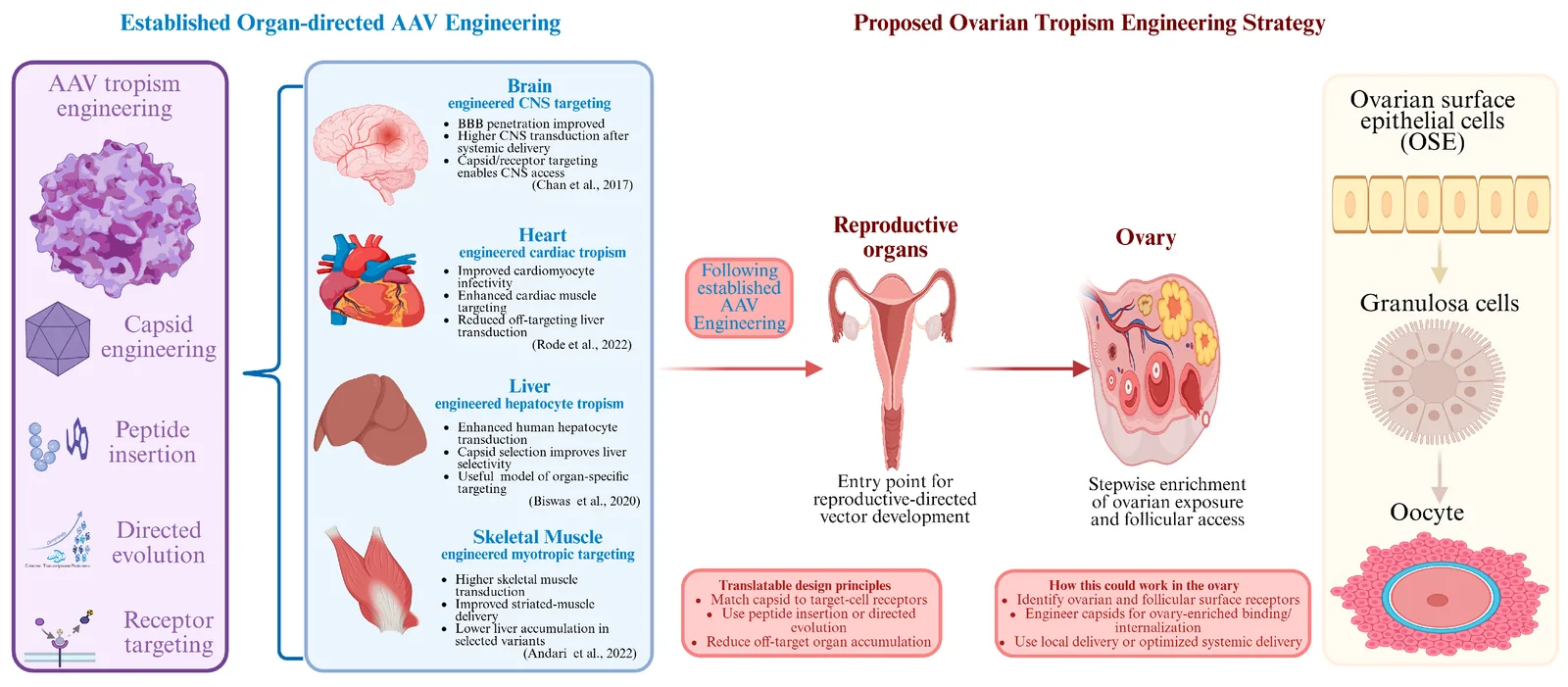
Conceptual framework for AAV tropism engineering toward ovarian cell targeting. How established AAV capsid engineering strategies for brain, heart, liver, and skeletal muscle targeting can guide ovarian vector development. Capsid engineering, peptide insertion, directed evolution, and receptor retargeting can redirect AAV tropism to specific tissues. Applied to reproductive gene therapy, delivery is progressively refined from reproductive organs to the ovary, then to surface epithelial cells, granulosa cells, and oocytes, combining ovary-enriched capsids with optimized routes and ovarian- or cell-type-specific promoters [63,64,65,66]. Created with BioRender.com.

**Figure 5:**
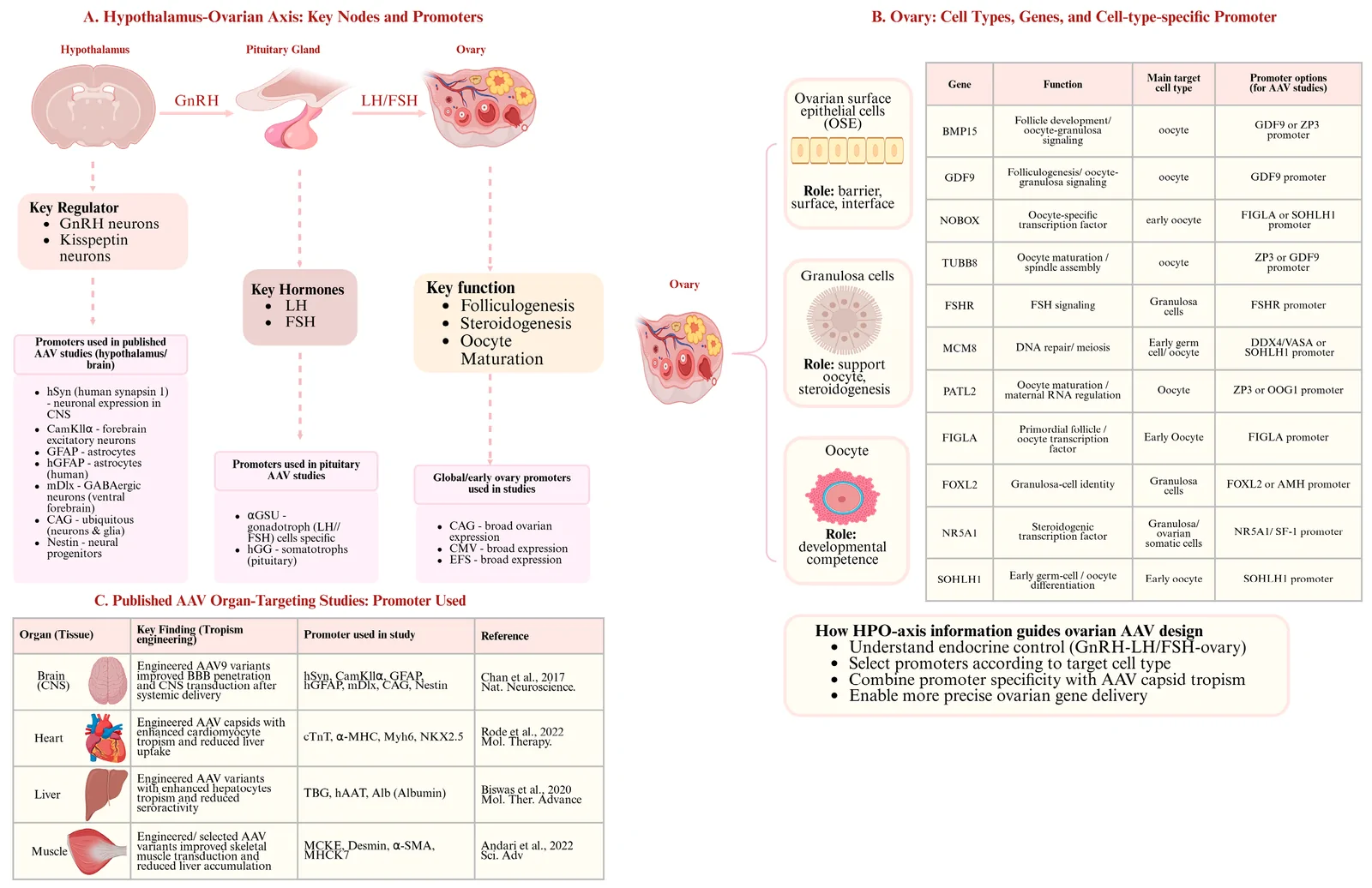
HPO-axis context and cell-type-specific promoter framework for AAV-mediated ovarian gene targeting. How the hypothalamus–pituitary–ovary axis can guide promoter selection for ovarian AAV gene therapy. The hypothalamus regulates ovarian function via GnRH, which stimulates pituitary LH and FSH release and controls folliculogenesis, steroidogenesis, and oocyte maturation. AAV studies in the brain, heart, liver, and skeletal muscle show that promoter choice can direct tissue- or cell-type-specific expression. Candidate genes are grouped by main target cell type—granulosa/somatic cells, surface epithelial interface, and oocytes—and, by matching each gene to an appropriate promoter and applying capsid engineering, may improve ovarian targeting while reducing off-target expression [63,64,65,66]. Created with BioRender.com.

**Table 1:** Regional distribution and occurrence of human female infertility genes.

Regions	Dominant Categories	Representative Genes	Interpretation	References
Republic of Korea/Japan	POI, ovarian reserve, hormonal signaling, DNA repair, oocyte maturation	BMP15, GDF9, NOBOX, FSHR, LHCGR, ESR1, INHA, MCM8, MCM9, TUBB8, TGFBR1, TGFBR3	Studies from Republic of Korea and Japan commonly emphasize ovarian reserve and hormone signaling genes, repeatedly highlighting BMP15, GDF9, FSHR, NOBOX, and MCM8/MCM9	[5,15,26]
China	Oocyte maturation arrest, POI/ovarian failure, meiosis/DNA repair, hormonal regulation	TUBB8, PATL2, WEE2, FOXL2, NOBOX, BMP15, GDF9, STAG3, MCM8, MCM9, MSH5, HFM1, CYP17A1, FSHR	China has large cohorts of individuals with infertility genetics; TUBB8 and PATL2 are repeatedly reported in oocyte maturation defects, while MCM8/MCM9/STAG3/MSH5/HFM1 are important in POI and meiotic failure	[27,28]
UAE/Middle East	Autosomal recessive meiotic defects, DNA repair, germ cell development, oocyte maturation	STAG3, HFM1, MCM8, MCM9, SOHLH1, SOHLH2, TUBB8	Consanguinity increases the detection of biallelic/recessive infertility genes, especially those involved in meiosis and germ cell development	[19,29]
USA	Multi-ethnic POI, DNA repair, oocyte maturation, maternal effect/embryo genes	FMR1, NOBOX, FIGLA, FOXL2, MCM8, MCM9, FANCM, TUBB8, PATL2, TLE6, BTG4, NLRP5, FSHR, STAG3	USA studies and commercial/research gene panels include a broad, multi-ethnic range, often combining POI, DNA repair, oocyte maturation, and early embryo genes	[30]
Europe	POI, endocrine infertility, meiosis, syndromic ovarian dysfunction	FMR1, BMP15, NOBOX, NR5A1, STAG3, PSMC3IP, GNRHR, KISS1R, TACR3, FOXL2, AARS2	European studies frequently report FMR1 premutation and NR5A1 in POI/endocrine infertility, with STAG3 and BMP15 also strongly represented	[19,31,32]

**Table 2:** Current therapeutic strategies for ovarian dysfunction and their relevance to AAV9-mediated gene therapy.

Therapeutic Strategy	Proposed Benefit	Major Limitation	Relevance to AAV9-Mediated Ovarian Gene Therapy	References
Lifestyle and nutritional intervention	May reduce systemic inflammation, oxidative stress, obesity-related metabolic stress, and environmental damage	Preventive/supportive only; cannot restore lost follicles or reverse advanced ovarian dysfunction	Provides baseline supportive care but does not directly correct molecular decline pathways	[2,37]
Antioxidant therapy	Reduces ROS and may improve mitochondrial function, oocyte quality, and granulosa cell survival	Effects are often modest, variable, dose-dependent, and unable to restore depleted follicle reserve	Supports the rationale for AAV9 delivery of antioxidant regulatory genes such as NRF2, SOD2, or CAT	[38,41,42]
Hormonal therapy and ART	Improves clinical fertility management, ovarian stimulation, oocyte retrieval, and menopausal symptom control	Does not reverse intrinsic oocyte damage, telomere attrition, senescence, or ovarian stromal deterioration	Highlights the need for mechanism-based therapy rather than only reproductive assistance	[39,40,43,44]
Mitochondrial-targeted therapy	May improve ATP production, mitochondrial quality control, and oocyte developmental competence	Mainly targets oocyte quality; broader ovarian niche and long-term safety remain unresolved	Supports AAV9 delivery of mitochondrial protective genes, such as SIRT3, PGC-1α, or TFAM	[3,45]
NAD^+^ boosters and geroprotective compounds	May enhance sirtuin activity, DNA repair, metabolism, and stress resistance	Clinical efficacy in ovarian impairment remains uncertain; systemic effects may vary	Provides mechanistic support for targeting sirtuin-related pathways using gene therapy	[46,47]
Platelet-rich plasma	May improve local growth factor signaling, angiogenesis, tissue repair, and follicular microenvironment	Protocols are not standardized; evidence is inconsistent; the molecular mechanism remains unclear	Serves as a local ovarian rejuvenation comparator for future AAV9 studies	[43,46]
Stem-cell-based therapy	May improve ovarian function through paracrine, anti-inflammatory, angiogenic, and regenerative effects	Cell source variability, safety concerns, tumor risk, immune response, and lack of standardization	Demonstrates interest in ovarian regeneration but lacks the gene-specific precision of AAV9	[48,49]
Stem-cell-derived extracellular vesicles	May deliver proteins, miRNAs, and signaling molecules that reduce apoptosis and inflammation	Cargo variability, dosing, manufacturing, and long-term efficacy remain unresolved	Supports the concept that molecular cargo delivery can modify ovarian dysfunction pathways	[48,50]
Rapamycin and other geroprotectors	May modulate mTOR, follicle activation, cellular senescence, and systemic decline pathways	Dose, timing, reproductive safety, and fertility balance are critical concerns	May be combined with AAV9 strategies but requires careful reproductive safety evaluation	[46,51]
Gene therapy	Enables sustained delivery of therapeutic genes targeting specific impairment mechanisms	Requires optimization of vector dose, targeting, immune safety, germline safety, and long-term expression	Represents a mechanism-based platform for correcting molecular drivers of ovarian deterioration	[6,7,8]

**Table 3:** Advantages, limitations, and design strategies for AAV9-mediated ovarian gene therapy.

AAV9 Features	Potential Advantage for Ovarian Therapy	Limitation or Safety Concern	Possible Design Strategy	References
Broad tissue tropism	Allows transduction of multiple tissues and may support systemic reproductive decline studies	Off-target transduction in liver, heart, muscle, nervous tissue, or other organs	Use local ovarian delivery, tissue-selective promoters, capsid engineering, and biodistribution analysis	[8,13,52,53]
Ability to transduce non-dividing cells	Suitable for ovarian somatic cells with limited proliferative activity, especially in aged tissue	Expression may be diluted in highly proliferative cells	Select appropriate target cells and evaluate the durability of expression	[8,52]
Long-term transgene expression	Useful for chronic decline processes requiring sustained molecular support	Difficult to stop expression if adverse effects occur	Use inducible promoters, self-limiting systems, or carefully selected promoter strength	[8,52]
Relatively low pathogenicity	Provides a favorable vector platform compared with many viral systems	AAV is not completely risk free and can still induce immune responses	Monitor anti-AAV antibodies, T-cell responses, cytokines, and local ovarian inflammation	[8,52,54]
Episomal persistence	Reduces the risk of insertional mutagenesis compared with integrating vectors	Rare integration events may still occur	Perform long-term safety monitoring and, when feasible, integration site analysis	[52,55]
Small packaging capacity	Encourages compact and efficient vector design	Limits the use of large genes full-length with large promoters/regulatory elements	Use compact promoters, optimized polyA signals, minimized regulatory elements, or smaller transgene variants	[8,52]
Evidence of ovarian cell transduction	Supports targeting granulosa and interstitial cells	Human ovarian transduction efficiency remains uncertain	Validate in aged mice, human ovarian tissue cultures, organoids, and larger animal models	[7,56]
Compatibility with local or systemic delivery	Allows flexible experimental design depending on the study goal	Systemic delivery may require higher doses and increase liver/immune toxicity	Compare direct ovarian, periovarian, intrabursal, intraperitoneal, and intravenous routes	[7,8,57]
Therapeutic gene flexibility	Can deliver genes targeting telomeres, mitochondria, oxidative stress, inflammation, and ECM remodeling	Some rejuvenation genes may increase the risk of tumorigenesis or physiological disruption	Select mechanism-specific genes and include tumor surveillance, endocrine analysis, and fertility testing	[57,58,59]
Translational experience in gene therapy	AAV platforms are widely used in preclinical and clinical gene therapy research	Reproductive and germline safety requirements are especially strict	Include ovarian biodistribution, oocyte safety, embryo development, offspring health, and long-term follow-up	[2,60]

**Table 4:** AAV packaging feasibility of selected ovarian dysfunction candidate genes.

Genes	NCBI Gene ID	CDS Size (kb)	Sources	AAV Feasibility
BMP15	Gene ID: 9210	1.18	NCBI RefSeq (NM_005448)	Suitable
GDF9	Gene ID: 2661	1.36	NCBI RefSeq (NM_005260)	Suitable
NOBOX	Gene ID: 79991	2.00	NCBI RefSeq (NM_001080413)	Suitable
TUBB8	Gene ID: 347688	1.34	NCBI RefSeq (NM_001271829)	Suitable
FSHR	Gene ID: 2492	2.10	NCBI RefSeq (NM_000145)	Suitable with cassette optimization
MCM8	Gene ID: 84524	2.50	NCBI RefSeq (NM_032485)	Suitable with cassette optimization
PATL2	Gene ID: 197135	1.44	NCBI RefSeq (NM_001145112)	Suitable
FIGLA	Gene ID: 6546	0.60	NCBI RefSeq (NM_001004311)	Suitable
FOXL2	Gene ID: 668	1.12	NCBI RefSeq (NM_023067)	Suitable
NR5A1	Gene ID: 2516	1.38	NCBI RefSeq (NM_004959)	Suitable
SOHLH1	Gene ID: 79600	1.26	NCBI RefSeq (NM_001101677)	Suitable

**Table 5:** Key preclinical evidence supporting the rationale for AAV9-mediated ovarian gene therapy.

Study Area	Main Finding	Relevance to This Review	References
AAV-mediated ovarian delivery and Kitl rescue	AAV vectors transduced ovarian granulosa cells and restored fertility-related defects in infertile mice	Provides direct proof-of-concept that ovarian somatic cells can be therapeutically targeted by AAV-mediated gene delivery	[7]
AAV9-mediated TERT anti-aging therapy	AAV9-TERT delayed aging-related phenotypes and increased longevity in adult and old mice without increased cancer incidence in the studied model	Supports the concept that AAV9 can deliver anti-aging genes and improve systemic decline phenotypes	[57,88,89]
AAV9-mediated MIS/AMH expression	AAV9 delivery of modified MIS/AMH regulated primordial follicle activation and protected ovarian reserve in mouse models	Demonstrates that AAV9-based gene delivery can modulate ovarian follicular dynamics	[90]
AAV8/AAV9 ovarian gene delivery	AAV8 and AAV9 delivered genes mainly to ovarian granulosa and interstitial cells	Supports the selection of AAV9 for ovarian somatic cell targeting and modulation of the follicular microenvironment	[56]
Mitochondrial dysfunction in ovarian dysfunction	Mitochondrial damage, reduced ATP production, and increased ROS are central mechanisms of oocyte and ovarian decline	Provides the rationale for mitochondrial candidate genes, including SIRT3, PGC-1α, and TFAM	[3,45,86]
Oxidative stress and granulosa cell dysfunction	Oxidative damage contributes to granulosa cell apoptosis, poor oocyte maturation, and impaired embryo development	Supports antioxidant regulatory targets, including NRF2, SOD2, CAT, and FOXO-related pathways	[84,85]
Cellular senescence and SASP in ovarian dysfunction	Senescent ovarian cells may produce inflammatory and matrix-remodeling factors that damage the follicular niche	Supports therapeutic strategies targeting telomere maintenance, DNA damage, inflammation, and senescence	[4,33]
Extracellular matrix remodeling and fibrosis	Decline-associated stromal fibrosis and ECM changes impair ovarian structure, vascular support, and follicle development	Supports investigation of ECM-modifying genes, such as HAS2/nmrHas2, but requires careful HA size and fibrosis analysis	[91,92]
AAV9 in reproductive disease models	AAV9-mediated delivery of therapeutic proteins for reproductive or ovarian disease produced measurable biological effects in vivo	Supports the feasibility of AAV9-based intervention in female reproductive tissues, although direct ovarian decline studies remain limited	[59]
Germline and offspring safety considerations	Some ovarian AAV studies suggest somatic ovarian targeting without detectable offspring genome integration under specific conditions	Highlights the need for rigorous analysis of germline transmission, embryo development, and offspring health in future ovarian AAV9 studies	[7,60,93]

**Table 6:** Regulatory landscape and translational considerations for AAV-mediated ovarian gene therapy across major regions.

Region	Authority/Framework	Gene Therapy Pathway	Key Regulatory Expectations	Ovarian AAV Consideration	Refs.
United States	FDA; CBER; Office of Therapeutic Products	Clinical development under an IND; licensed products regulated as biologics	Product quality and CMC; pharmacology/toxicology; biodistribution; dose; immunogenicity; clinical safety; long-term follow-up	Distinguish somatic ovarian transduction from germ cell exposure; assess reproductive outcomes, systemic exposure, persistence, and delayed effects	[96,102,103]
European Union/EEA	EMA; Committee for Advanced Therapies; GTMP/ATMP framework	In vivo gene therapies evaluated as GTMPs under the ATMP framework	Quality and potency; proof-of-concept; biodistribution; toxicology; dose; immunogenicity; clinical safety; risk management; pharmacovigilance	Establish a germline risk strategy and demonstrate predominant expression in ovarian somatic cells rather than oocytes or germ cells	[97,104]
United Kingdom	MHRA; ATMP framework under UK medicines legislation	MHRA clinical trial authorization and marketing authorization	Risk-based CMC; manufacturing controls; non-clinical and clinical evidence; dose and safety rationale; GMO oversight when applicable	Assess target specificity, gonadal and systemic biodistribution, expression duration, reproductive toxicity, and unintended oocyte exposure	[98,105]
Japan	PMDA; Ministry of Health, Labour and Welfare; regenerative medical-product framework	Gene therapy guidance and PMDA scientific consultation	Quality/CMC; target specificity; non-clinical pharmacology and safety; clinical planning; target and non-target toxicity; GMO requirements	Quantify non-target transduction and determine whether ovarian somatic targeting can be separated from oocyte or germ cell exposure	[99,106]
Republic of Korea	MFDS: advanced biopharmaceutical and gene therapy framework	MFDS regulation of gene therapy and advanced biopharmaceutical products	Product quality; pharmacology/toxicology; biodistribution; vector safety; clinical rationale; risk-based development	Assess ovarian and systemic biodistribution, reproductive safety, persistence, and unintended oocyte or germ cell exposure	[100,107]
China	NMPA; Center for Drug Evaluation	Commercial IND pathway; separate investigator-initiated pathway may apply	CMC; pharmacology; dose response; biodistribution; toxicology; clinical protocol; long-term risk assessment	Confirm intended ovarian targeting and assess systemic deposition, germ cell exposure, reproductive effects, persistence, and transmission risk	[107]
Australia	TGA; Office of the Gene Technology Regulator when applicable	Clinical Trial Notification or Clinical Trial Approval pathway; EMA-aligned GTMP guidance	Quality/CMC; proof-of-concept; biodistribution; toxicology; clinical safety and efficacy; risk-based and GMO oversight	Distinguish somatic ovarian targeting from germ cell exposure and assess gonadal biodistribution and reproductive consequences	[101,104]

## Data Availability

No new data were created or analyzed in this study. Data sharing does not apply to this article.

## Supplementary Materials

The following supporting information can be downloaded at: https://www.mdpi.com/article/10.3390/ijms27198516/s1.

## Abbreviations

The following abbreviations are used in this manuscript:

AAV	Adeno-associated virus
AAV9	Adeno-associated virus serotype 9
ALT	Alanine aminotransferase
ART	Assisted reproductive technology
AST	Aspartate aminotransferase
ATMP	Advanced therapy medicinal product
ATP	Adenosine triphosphate
BBB	Blood–brain barrier
CAG	Cytomegalovirus enhancer/chicken β-actin promoter
CBER	Center for Biologics Evaluation and Research
CDS	Coding sequence
CMC	Chemistry, manufacturing, and controls
CMV	Cytomegalovirus
CoQ10	Coenzyme Q10
DNA	Deoxyribonucleic acid
EEA	European Economic Area
ECM	Extracellular matrix
EMA	European Medicines Agency
FDA	United States Food and Drug Administration
FSH	Follicle-stimulating hormone
GMO	Genetically modified organism
GnRH	Gonadotropin-releasing hormone
GTMP	Gene therapy medicinal product
HA	Hyaluronic acid
HPO	Hypothalamic–pituitary–ovarian
IDDB	Human Infertility Disease Database
IND	Investigational New Drug
IVF	In vitro fertilization
LH	Luteinizing hormone
MFDS	Ministry of Food and Drug Safety
MHRA	Medicines and Healthcare products Regulatory Agency
NAD^+^	Nicotinamide adenine dinucleotide
NCBI	National Center for Biotechnology Information
NF-κB	Nuclear factor kappa B
NMPA	National Medical Products Administration
OMIM	Online Mendelian Inheritance in Man
PGC-1α	Peroxisome proliferator-activated receptor gamma coactivator 1-alpha
PMDA	Pharmaceuticals and Medical Devices Agency
POI	Primary ovarian insufficiency
PRP	Platelet-rich plasma
RNA	Ribonucleic acid
ROS	Reactive oxygen species
SASP	Senescence-associated secretory phenotype
TGA	Therapeutic Goods Administration
TGF-β	Transforming growth factor beta
WPRE	Woodchuck hepatitis virus posttranscriptional regulatory element
AMED	Japan Agency for Medical Research and Development
ASRM	American Society for Reproductive Medicine
CIHR	Canadian Institutes of Health Research
MRC	Medical Research Council
NHMRC	National Health and Medical Research Council
NIA	National Institute on Aging
NICHD	Eunice Kennedy Shriver National Institute of Child Health and Human Development
NIH	National Institutes of Health
UKRI	UK Research and Innovation
