# Supplementary material for: AAV9 Gene Therapy for Ovarian Dysfunction and Reproductive Aging: Molecular Mechanisms, Therapeutic Targets, and Translational Perspectives
Source: Int J Mol Sci. 2026 Sep 24;27(19):8516. doi: 10.3390/ijms27198516 (PMC13665288; doi:10.3390/ijms27198516)
Supplement: Supplementary file 1 [file ijms-27-08516-s001.zip › ijms-4546058-supplementary.pdf]

AAV9 Gene Therapy for Ovarian Dysfunction and Reproductive Aging: Molecular Mechanisms, Therapeutic Targets, and Translational Perspectives

Safeer Ullah[1,#], Imran Ullah[1,2,3,#], Beshoy Sameh Fawzy Khalil[1], Zhaojun Geng[1], Jihyo Kim[1], Seung-Eun Lee[1], Xiaochun Wu[1], Kamran Saeed[1,2], Abdus Saboor Shah[6], Muhammad Idrees[4†], Il-Keun Kong[1,2,4,5*†]

[1]Division of Applied Life Science (BK21 Four), Gyeongsang National University, Jinju 52828, Gyeongnam Province, Republic of Korea.

[2]Institute of Agriculture and Life Science, Gyeongsang National University, Gyeongnam Province, Republic of Korea.

[3]Department of Biochemistry, Faculty of Biological Sciences, Quaid-i-Azam University, 45320, Islamabad, Pakistan

[4]The King Kong Corp. Ltd., Gyeongsang National University, Jinju 52828, Gyeongnam Province, Republic of Korea.

[5]Division of Animal Science, Gyeongsang National University, Jinju 52828, Republic of Korea

[6]Department of Chemistry, University of Science and Technology, Bannu, Khyber-Pakhtunkhwa, Pakistan

[#]These authors contributed equally

[†]Correspondence: Il-Keun Kong (ikong7900@gmail.com/ ikong@gnu.ac.kr) Tel.: +82-55-772-1942

[†]Co-correspondence: Muhammad Idrees (idrees1600@gmail.com)

Keywords: *AAV9; ovarian dysfunction; female infertility; gene therapy; reproductive aging;*

SUPPLEMENTARY DATA

Supplementary Data Table S1. Classification of reported female infertility genes in Human Infertility Disease Database (IDDB)

Disease type/category	No. of genes reported	Interpretation for this review	References
**Female infertility**	136	Total female infertility genes considered from the IDDB resource.	[1,2]
**Endocrine diseases**	47	Genes affecting reproductive hormone synthesis, secretion, receptor signaling, and hypothalamic-pituitary-gonadal regulation.	
**Ovarian dysfunction**	60	Genes affecting ovarian development, follicular reserve, granulosa/oocyte interaction, folliculogenesis, and POI.	
**Infertility due to oocyte factors**	10	Genes affect oocyte maturation, fertilization, maternal-effect function, and early embryo development.	
**Unspecified syndrome with female infertility**	19	Genes causing syndromic or broader developmental conditions where infertility is one clinical feature.	

Supplementary Data Table S2. Major primary ovarian insufficiency (POI) and ovarian failure genes relevant to ovarian dysfunction

Genes	Major function/relevance	References
**BMP15**	Oocyte-secreted TGF-beta superfamily growth factor controlling granulosa-cell function and follicle development.	[1,2,3,4]
**GDF9**	Oocyte-derived growth factors are essential for early folliculogenesis and oocyte-granulosa signaling.	
**FOXL2**	Ovarian differentiation and granulosa-cell identity transcription factor.	
**NOBOX**	Oocyte-specific homeobox transcription factor regulating primordial follicle formation and oocyte gene expression.	
**FIGLA**	Basic helix-loop-helix transcription factor required for primordial follicle formation and zona pellucida gene expression.	
**FSHR**	Follicle-stimulating hormone receptor is required for follicle growth and granulosa-cell endocrine response.	

**NR5A1 (SF-1)**	Steroidogenic transcription factor involved in gonadal development and steroid hormone regulation.	
**SOHLH1**	Germ-cell transcription factor regulating oocyte differentiation and early folliculogenesis.	
**LHX8**	Oocyte transcription factor involved in oocyte survival and follicle development.	
**FMR1**	Fragile-X premutation gene associated with increased risk of POI.	
**EIF4ENIF1**	Oocyte translational regulator linked with ovarian insufficiency.	
**PGRMC1**	Progesterone receptor membrane component involved in ovarian cell survival and steroid signaling.	
**AARS2**	Mitochondrial alanyl-tRNA synthetase linked with syndromic ovarian insufficiency.	
**BNC1**	Germ-cell transcription factor associated with ovarian and oocyte development.	

Supplementary Data Table S3. Major meiosis and DNA repair genes associated with ovarian dysfunction

Genes	Major functions/relevances	References
**STAG3**	Meiotic cohesin component required for chromosome cohesion and meiotic progression.	[1,2,4,5]
**SMC1B**	Meiotic cohesin complex component maintaining sister-chromatid cohesion.	
**REC8**	Meiotic cohesion subunit is essential for chromosome segregation.	
**SYCE1**	Synaptonemal complex protein required for meiotic chromosome pairing.	
**HFM1**	DNA helicase is involved in meiotic recombination and crossover formation.	
**MSH4**	Mismatch-repair protein required for meiotic recombination.	
**MSH5**	Meiotic recombination and crossover-associated DNA repair factor.	
**MCM8**	DNA repair factor involved in homologous recombination during meiosis.	
**MCM9**	Partner of MCM8 is required for homologous recombination repair and ovarian maintenance.	
**PSMC3IP (HOP2)**	Meiotic recombination mediator acting with DMC1/RAD51-related pathways.	
**SPIDR**	DNA double-strand break repair protein.	
**RFWD3**	DNA repair protein involved in replication stress and homologous recombination.	
**SLX4**	DNA repair scaffold protein.	

**HELQ**	Helicase involved in genome stability and DNA repair.	
**KASH5**	Meiotic chromosome movement and pairing factor.	

Supplementary Data Table S4. Major oocyte maturation and fertilization genes associated with female infertility

Genes	Major functions/relevances	References
**TUBB8**	Human oocyte beta-tubulin isotype required for meiotic spindle assembly.	[1,2,6]
**WEE2**	Oocyte meiotic cell-cycle kinase required for fertilization and egg activation.	
**TLE6**	Subcortical maternal-complex protein required for early embryonic development.	
**PATL2**	Oocyte RNA-binding/regulatory protein associated with oocyte maturation arrest.	
**PANX1**	Oocyte membrane channel variants can cause oocyte death and infertility.	
**ZAR1**	Maternal-effect gene involved in oocyte-to-embryo transition.	
**BTG4**	Maternal mRNA clearance factor required for zygotic genome activation.	
**CDC20**	Cell-cycle regulator required for meiotic progression.	
**CCNB3**	Cyclin involved in meiotic cell-cycle control.	
**CENPE**	Kinetochore motor protein involved in chromosome alignment.	

Supplementary Data Table S5. Major hormonal signaling and endocrine regulation genes associated with female infertility

Genes	Major function/relevance	References
**AMH**	Marker and regulator of ovarian reserve and primordial follicle activation.	[1,2,7]
**AMHR2**	Anti-Mullerian hormone receptor involved in follicle recruitment regulation.	
**LHB**	Luteinizing hormone beta subunit.	
**LHCGR**	LH/chorionic-gonadotropin receptor required for ovulation and luteinization.	
**CYP17A1**	Steroidogenic enzyme for androgen/estrogen precursor synthesis.	
**CYP19A1 (Aromatase)**	Enzyme required for estrogen biosynthesis.	
**CYP21A2**	Steroidogenesis enzyme involved in adrenal/ovarian steroid balance.	

**STAR**	Steroidogenic acute regulatory protein required for cholesterol transfer into mitochondria.	
**KISS1**	Kisspeptin ligand regulating GnRH secretion.	
**KISS1R**	Kisspeptin receptor required for reproductive axis activation.	
**GNRH1**	Gonadotropin-releasing hormone.	
**GNRHR**	GnRH receptor mediating pituitary gonadotropin release.	
**TAC3**	Neurokinin B ligand involved in GnRH pulse regulation.	
**TACR3**	Neurokinin B receptor involved in reproductive endocrine control.	

Supplementary Data Table S6. Major folliculogenesis and ovarian development genes associated with ovarian dysfunction

Genes	Major function/relevance	References
**BMPR2**	BMP signaling receptor involved in follicular development.	[1,2,4]
**WT1**	Gonadal development transcription factor.	
**KHDRBS1**	RNA-binding protein involved in granulosa-cell biology.	
**HSD17B1**	Estrogen metabolism enzyme.	
**HMMR**	Granulosa-cell proliferation and extracellular-matrix-associated factor.	
**ALOX12**	Ovulation-related lipid signaling enzyme.	
**MST1R**	Cell-signaling receptor involved in follicle development.	
**PPM1B**	Protein phosphatase linked with ovulatory regulation.	
**ZP3**	Zona pellucida glycoprotein required for fertilization.	
**PRDM1**	Germ-cell specification regulator.	

Supplementary Data Table S7. Region-specific candidate genes prioritized for future AAV-based gene supplementation studies

Regions	Most characteristic genes selected for future AAV-based gene supplementation studies	References
**South Korea**	BMP15, GDF9, TUBB8, FSHR, MCM8	[1,4,5,8,9]
**China**	TUBB8, PATL2, MCM8, MCM9, FSHR	
**USA**	MCM8, TUBB8, PATL2, FIGLA, MCM9, FOXL2	

**UAE / Gulf**	STAG3, MCM8, TUBB8, HFM1	
**Europe**	BMP15, STAG3, NOBOX, FOXL2, NR5A1	

Supplementary Data Table S8. Representative funding agencies and programs relevant to ovarian biology, reproductive aging, and gene-therapy development.

Region	Funding agency/program	Scope and mechanism	Relevance to ovarian biology / reproductive aging	Potential relevance to AAV-mediated ovarian gene therapy	Refs.
**United States**	NIH: National Institute on Aging (NIA) and NICHD Fertility and Infertility Branch	Basic, mechanistic, clinical, and translational research on aging, fertility, and reproductive biology	Ovarian aging, POI, follicular decline, fertility preservation, and reproductive lifespan	Mechanism-driven studies with proof-of-concept, biodistribution, safety, and a realistic translational plan	[10], [11]
**United States / international**	ASRM Research Institute - Discovery & Innovation Grant	Investigator-led basic and clinically relevant reproductive-medicine research	Ovarian biology, fertility, and underexplored reproductive-health questions	Early proof-of-concept, ovarian tropism, target validation, or fertility-preservation studies	[12]
**European Union**	Horizon Europe - Cluster 1: Health	Collaborative research in health, regenerative medicine, ATMPs, biomedical technologies, and regulatory science	Multidisciplinary women's-health and reproductive-aging projects aligned with specific calls	Best suited to consortia studying vector engineering, biodistribution, safety, manufacturing, or regulatory science	[13]
**United Kingdom**	UKRI Medical Research Council - Proof of Concept	Translational de-risking of promising therapeutics and other healthcare interventions	Ovarian-aging or infertility interventions that have progressed	Proof-of-concept development involving dose, delivery, safety, biomarkers, or	[14]

			beyond discovery	manufacturability	
**United Kingdom / eligible international settings**	Wellcome Discovery Awards	Long-term, multidisciplinary research intended to produce major advances in human health understanding	Fundamental reproductive-aging mechanisms, ovarian biology, and enabling delivery platforms	Appropriate for transformative mechanistic or platform advances rather than incremental vector testing	[15]
**Japan**	AMED - Project for Regenerative Medicine and Cell and Gene Therapies	Cell and gene therapy from basic research through translation, manufacturing, vector production, and commercialization	Mechanistically defined ovarian disorders with preclinical or translational potential	Vector platforms, proof-of-concept, manufacturing, and translational development for ovarian AAV	[16]
**South Korea**	NRF - Bio & Medical Technology Development Program	Advanced biotechnology, new drugs, precision medicine, and next-generation medical technologies	Reproductive biotechnology, ovarian-aging mechanisms, target discovery, and therapeutic platforms	Vector engineering, mechanistic validation, preclinical safety, and advanced therapeutic development	[17]
**Canada**	CIHR - Project Grant Program	Fundamental and applied health research from discovery through translation and commercialization	Ovarian aging, infertility, reproductive health, and women's health	Mechanism-driven research supported by biological rationale, feasibility, safety, and patient relevance	[18]
**Australia**	NHMRC - Reframing Women's Health 2026	Research addressing major women's-health gaps through mechanisms,	Ovarian aging and clinically meaningful reproductive-health questions	Ovarian AAV as part of a broader women's-health study rather	[19]

		interventions, and translation		than a vector-only project	

Funding programs are representative rather than exhaustive. Most programs are not dedicated specifically to ovarian AAV gene therapy; they support one or more relevant domains such as ovarian biology, reproductive aging, women's health, gene therapy, regenerative medicine, or translational therapeutics. Program scope, eligibility, and call status should be verified at the time of submission.

Supplementary Figure

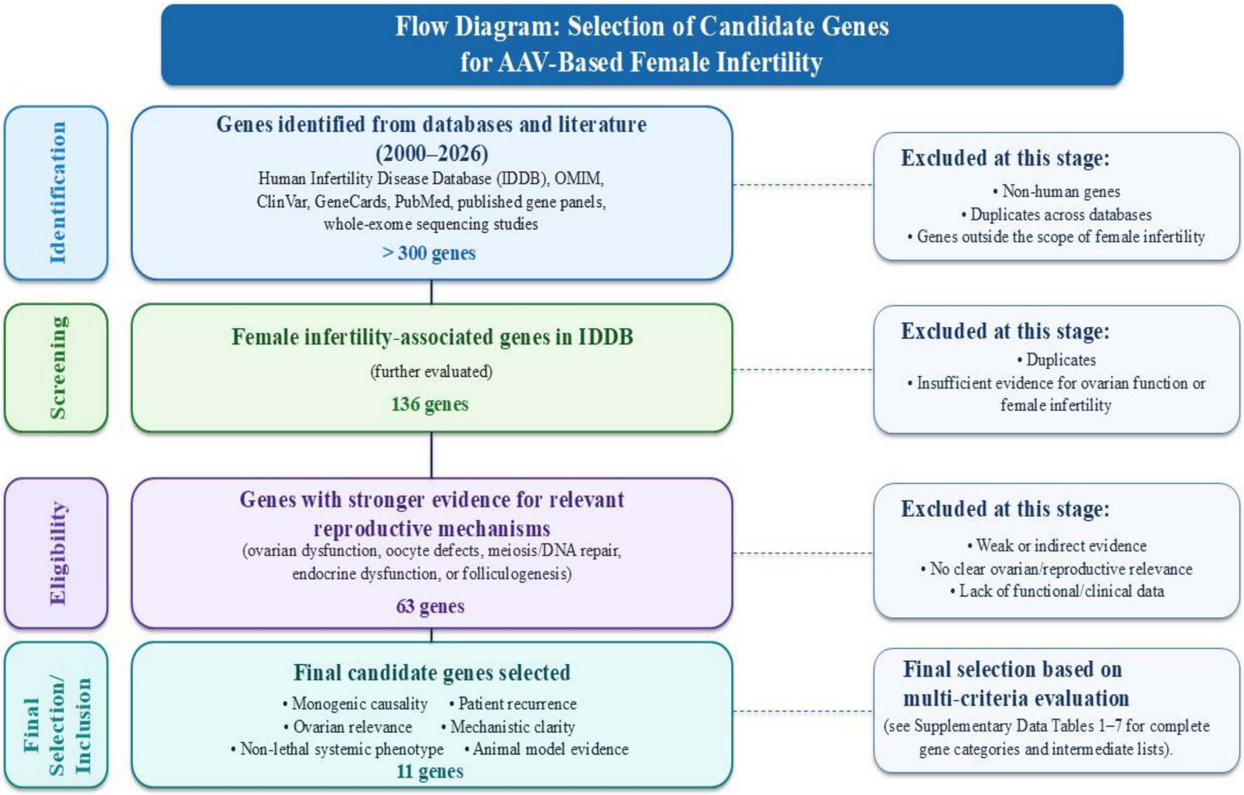

Supplementary Figure S1. Flow diagram of candidate-gene prioritization for AAV-based approaches to female infertility. More than 300 genes were identified from databases and the literature; 136 female infertility-associated genes were evaluated, 63 with stronger evidence of relevance to reproductive mechanisms were shortlisted, and 11 were prioritized based on monogenic causality, ovarian relevance, clinical recurrence, and animal-model evidence.

REFERENCES

[1] C. Friedrich and F. Tüttelmann, “Genetics of female and male infertility,” *Medizinische Genetik*, vol. 36, no. 3, pp. 161–170, Sep. 2024, doi: 10.1515/medgen-2024-2040.

[2] S. Caburet *et al.*, “Mutant Cohesin in Premature Ovarian Failure,” *N. Engl. J. Med. 2014;370(10):943–949. DOI: 10.1056/NEJMoa1309635.*

[3] M. K. Herndon and J. H. Nilson, “Maximal expression of FOXL2 in pituitary gonadotropes requires ovarian hormones,” *PLoS One*, vol. 10, no. 5, May 2015, doi: 10.1371/journal.pone.0126527.

[4] M. I. Stamou and N. A. Georgopoulos, “Kallmann syndrome: phenotype and genotype of hypogonadotropic hypogonadism,” *Metabolism*, vol. 86, pp. 124–134, Sep. 2018, doi: 10.1016/j.metabol.2017.10.012.

[5] M. Zorrilla and A. N. Yatsenko, “The Genetics of Infertility: Current Status of the Field,” *Curr. Genet. Med. Rep.*, vol. 1, no. 4, pp. 247–260, Dec. 2013, doi: 10.1007/s40142-013-0027-1.

[6] G. Lu, Y. Y. Zhu, H. X. Li, Y. L. Yin, J. Shen, and M. H. Shen, “Effects of acupuncture treatment on microRNAs expression in ovarian tissues from Tripterygium glycoside-induced diminished ovarian reserve rats,” *Front. Genet.*, vol. 13, Sep. 2022, doi: 10.3389/fgene.2022.968711.

[7] J. Shen *et al.*, “Genetic etiologic analysis in 74 Chinese Han women with idiopathic premature ovarian insufficiency by combined molecular genetic testing,” *J. Assist. Reprod. Genet.*, vol. 38, no. 4, pp. 965–978, 2021, doi: 10.1007/s10815-021-02083-7.

[8] D. Li *et al.*, “Three Novel Mutations in TUBB8 Cause Female Infertility Due to Multiple Morphological Abnormalities of the Oocyte and Early Embryo,” *Reproductive Sciences*, vol. 32, no. 6, pp. 1813–1824, 2025, doi: 10.1007/s43032-025-01844-4.

[9] L. Biswas, K. Tyc, W. El Yakoubi, K. Morgan, J. Xing, and K. Schindler, “Meiosis interrupted: the genetics of female infertility via meiotic failure,” Reproduction 2021, 161, R13–R35. https://doi.org/10.1530/REP-20-0422.

[10] National Institute on Aging, “Mechanisms of Sex Differences and Reproductive Aging Program,” National Institute on Aging.https://www.nia.nih.gov/research/dab/translational-research-branch-and-programs (accessed on 14 September 2026).

[11] Eunice Kennedy Shriver National Institute of Child Health and Human Development, “Fertility and Infertility Branch (FIB),” NICHD. Available online: https://www.nichd.nih.gov/about/org/der/branches/fib (accessed on 14 September 2026).

[12] ASRM Research Institute, “ASRM Discovery & Innovation Grant,” American Society for Reproductive Medicine. Available online: https://connect.asrm.org/asrmresearch/rfp/di-grant-opportunities/asrm-di-grant-guidelines (accessed on 14 September 2026).

[13] European Health and Digital Executive Agency, “2026 Horizon Europe ‘Health’ calls for proposals,” European Health and Digital Executive Agency (HaDEA). Available online: https://hadea.ec.europa.eu/news/2026-horizon-europe-health-calls-proposals-2026-02-12_en (accessed on 14 September 2026).

[14] UK Research and Innovation, “UKRI translation: MRC proof of concept: stage two,” UK Research and Innovation (UKRI). Available online: https://www.ukri.org/opportunity/mrc-proof-of-concept-stage-two/ (accessed on 14 September 2026).

[15] Wellcome, “Wellcome Discovery Awards.” Available online: https://wellcome.org/research-funding/schemes/wellcome-discovery-awards(accessed on 14 September 2026).

[16] Japan Agency for Medical Research and Development, “Project for Regenerative Medicine and Cell and Gene Therapies,” Japan Agency for Medical Research and Development (AMED). Available online: https://www.amed.go.jp/en/program/index03.html (accessed on 14 September 2026).

[17] National Research Foundation of Korea (NRF), “Bio & Medical Technology Development Program.” Available online: https://eng.nrf.re.kr/page/5301bb32-ad33-4031-8de1-a8b3275956a2?bizNo=3 (accessed on 14 September 2026).

[18] Canadian Institutes of Health Research, “Project Grant Program,” Canadian Institutes of Health Research (CIHR). Available online: https://cihr-irsc.gc.ca/e/49051.html (accessed on 14 September 2026).

[19] National Health and Medical Research Council (NHMRC), “Targeted Call for Research: Reframing Women’s Health 2026.” Available online: https://www.nhmrc.gov.au/funding/find-funding/targeted-call-research-reframing-womens-health-2026 (accessed on 14 September 2026).
